# Engineering of a GLP-1 analogue peptide/anti-PCSK9 antibody fusion for type 2 diabetes treatment

**DOI:** 10.1038/s41598-018-35869-4

**Published:** 2018-12-03

**Authors:** Matthieu Chodorge, Anthony J. Celeste, Joseph Grimsby, Anish Konkar, Pia Davidsson, David Fairman, Lesley Jenkinson, Jacqueline Naylor, Nicholas White, Jonathan C. Seaman, Karen Dickson, Benjamin Kemp, Jennifer Spooner, Emmanuel Rossy, David C. Hornigold, James L. Trevaskis, Nicholas J. Bond, Timothy B. London, Andrew Buchanan, Tristan Vaughan, Cristina M. Rondinone, Jane K. Osbourn

**Affiliations:** 1 Antibody Discovery and Protein Engineering, MedImmune, Cambridge, UK; 2 Biosuperiors, MedImmune, Gaithersburg, USA; 3 Biosuperiors, MedImmune, Cambridge, UK; 4 Cardiovascular, Renal and Metabolic diseases, MedImmune, Gaithersburg, USA; 5 Cardiovascular, Renal and Metabolic diseases, MedImmune, Cambridge, UK; 6 Cardiovascular and Metabolic diseases, Innovative Medicines and Early Development Biotech unit, Translational Science, AstraZeneca R&D, Gothenburg, Sweden; 7 Clinical Pharmacology and DMPK, MedImmune, Cambridge, UK; 8 Biopharmaceutical Development, MedImmune, Cambridge, UK

## Abstract

Type 2 diabetes (T2D) is a complex and progressive disease requiring polypharmacy to manage hyperglycaemia and cardiovascular risk factors. However, most patients do not achieve combined treatment goals. To address this therapeutic gap, we have developed MEDI4166, a novel glucagon-like peptide-1 (GLP-1) receptor agonist peptide fused to a proprotein convertase subtilisin/kexin type 9 (PCSK9) neutralising antibody that allows for glycaemic control and low-density lipoprotein cholesterol (LDL-C) lowering in a single molecule. The fusion has been engineered to deliver sustained peptide activity *in vivo* in combination with reduced potency, to manage GLP-1 driven adverse effects at high dose, and a favourable manufacturability profile. MEDI4166 showed robust and sustained LDL-C lowering in cynomolgus monkeys and exhibited the anticipated GLP-1 effects in T2D mouse models. We believe MEDI4166 is a novel molecule combining long acting agonist peptide and neutralising antibody activities to deliver a unique pharmacology profile for the management of T2D.

## Introduction

Type 2 diabetes (T2D) is a chronic illness of pandemic proportions affecting over 400 million people worldwide (www.idf.org). It is characterized by hyperglycaemia, resulting from the progressive loss of pancreatic β-cell function in the presence of insulin resistance. Atherosclerotic cardiovascular disease (ASCVD) is the leading cause of morbidity and mortality in T2D patients and is the largest contributor of direct and indirect costs of diabetes^1^. Treatment strategies are often multifactorial and target central ASCVD risk factors including poor glycaemic control, dyslipidaemia, obesity and hypertension. However less than 10% of patients achieve combined target goals^2^. To address this treatment gap, we engineered a proprotein convertase subtilisin/kexin type 9 (PCSK9) neutralizing monoclonal antibody (mAb) fused to a glucagon-like peptide-1 (GLP-1) receptor (GLP-1R) agonist peptide to deliver low-density lipoprotein cholesterol (LDL-C) lowering capabilities combined with glycaemic and weight control in a single molecule.

GLP-1 is a peptide hormone notably produced by the intestinal enteroendocrine L-cells of the gut. It is secreted following nutrient ingestion, resulting in enhanced glucose-stimulated insulin secretion (GSIS) from the pancreatic β-cells as well as delayed gastric emptying and enhanced satiety, making it an attractive target for the treatment of T2D and obesity^3^. GLP-1 is rapidly degraded by the dipeptidyl peptidase-4 enzyme (DPP-4) requiring the development of DPP-4 resistant peptide analogues to generate robust efficacy. Prolonged activity, compatible with weekly dosing for improved patient convenience, was further achieved using slow release formulations or genetic fusion with a long half-life carrier protein as an antibody Fc or serum albumin^4^. GLP-1 receptor agonists (GLP-1RA) effectively reduce glycated haemoglobin (HbA1c), lower body weight and provide cardiovascular risk reductions^5,6^. To improve the cardiovascular benefits of GLP-1RAs, we elected to target LDL-C lowering by fusing a GLP-1 analogue peptide to a neutralizing PCSK9 monoclonal antibody.

PCSK9 is a serum protein that binds to the LDL receptor and promotes its degradation, resulting in increased levels of LDL-C in circulation^7^. Anti-PCSK9 antibodies, such as evolocumab and alirocumab, block the PCSK9 interaction with LDL receptor and have demonstrated robust LDL-C reductions in hypercholesterolemic patients^8^. In addition, PCSK9 antibodies have shown an additive effect with statin therapy^9^ and reduced the risk of cardiovascular events^10^.

Here we describe the generation and preclinical evaluation of MEDI4166, a dual anti-PCSK9 antibody fused with a GLP-1 analogue agonist peptide, with the potential to provide significant benefit to T2D patients with high cardiovascular risk. As proof of concept that PCSK9/GLP-1 peptide-antibody molecules can deliver dual pharmacology, we investigated a panel of N-terminal genetic fusions of a DPP-4-resistant GLP-1 analogue with different PCSK9 antibodies and linkers. Numerous challenges had to be addressed through peptide engineering and pharmacokinetic-pharmacodynamic (PK-PD) modelling to develop MEDI4166. These included: improving the *in vivo* stability of the GLP-1 analogue peptide which was significantly shorter than the antibody moiety; tuning the potency at GLP-1 receptor to achieve efficient and safe GLP-1 activity at doses compatible with strong PCSK9 suppression; and minimising the propensity of the fusion molecule to aggregate.

## Results

### Dual function of GLP-1 receptor agonist peptide and anti-PCSK9 antibody fusions

**Figure 1:**
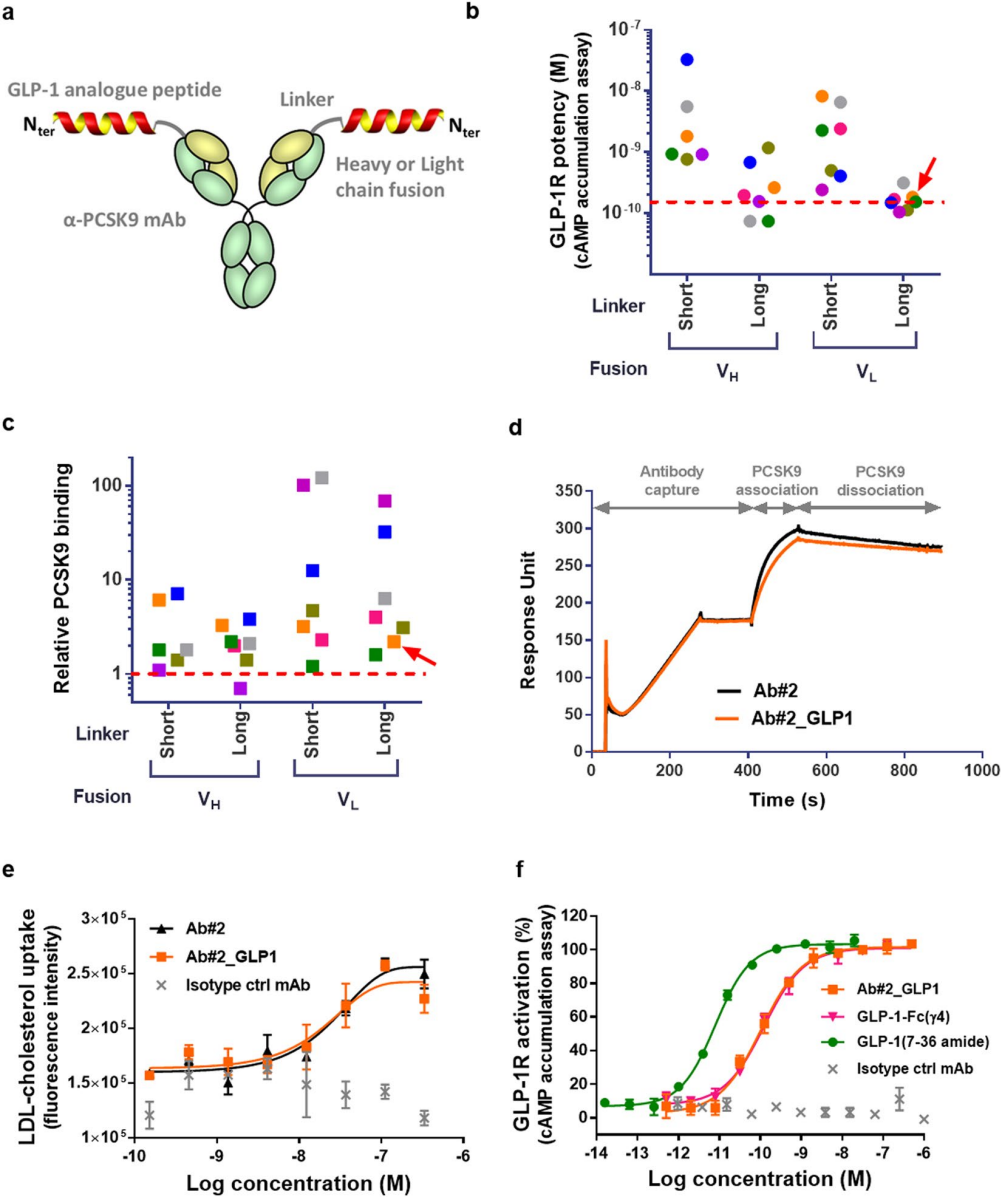
Dual activity of GLP-1 receptor agonist peptide and anti-PCSK9 antibody fusions. (**a**) Schematic representation of GLP-1 analogue peptide anti-PCSK9 antibody fusion. (**b**) Potency at human GLP-1R in CHO transfected cells of the 27 tested peptide antibody fusions in the cAMP accumulation assay using anti-PCSK9 Ab#1 (pink), Ab#2 (orange), Ab#3 (khaki), Ab#4 (green), Ab#5 (blue), Ab#6 (purple) and Ab#7 (grey). Potency of the reference compound GLP-1-Fc(γ4) is shown with a dashed red line. The red arrow shows the fusion chosen for in-depth characterisation. (**c**) Relative PCSK9 binding of the 27 tested peptide antibody fusions in the biochemical epitope competition assays compared to the parental anti-PCSK9 mAbs. A relative binding of 1, shown with a dashed red line, is corresponding to the parent antibody EC_50_. (**d**) Surface plasmon resonance (SPR) sensorgrams for binding of recombinant human PCSK9 to Ab#2 (black) and peptide antibody fusion Ab#2_GLP1 (orange). The graph illustrates data of a typical experiment done 8 times independently. (**e**) Uptake of fluorescently labelled LDL-cholesterol by HepG2 cells treated with 45 nM of recombinant human PCSK9 and Ab#2 (black), peptide antibody fusion Ab#2_GLP1 (orange) or isotype control mAb (grey), (n = 2) (**f**) Activation of human GLP-1R as % of maximum GLP-1(7–36) amide peptide response in CHO transfected cells using cAMP accumulation assay following treatment with Ab#2_GLP1 (orange), GLP-1-Fc(γ4) (pink), GLP-1(7–36) amide peptide (green) or isotype control mAb (grey), (n = 2). For (**b**) and (**c**), values are shown as mean of n = 2. For (**e**) and (**f**), values are presented as mean (±SD) of a typical experiment performed independently at least in duplicate.

Based on the critical requirement for a free N-terminus on the GLP-1 peptide for receptor activation^11^, we genetically fused a DPP-4-resistant GLP-1R agonist peptide, similar to the one used in the Fc fusion molecule LY2189265^12^, to either the heavy or light chain variable domain of neutralising anti-PCSK9 antibodies (Fig. 1a). We investigated a panel of seven antibodies, from multiple origins (Supplementary Table 1) and of diverse variable chain sequences (Supplementary Table 2), fused to the peptide with either a short (GGSA) or a long ([G_4_S]_3_A) amino acid linker. The fusion constructs were expressed, purified and tested for both human GLP-1R potency in a cyclic adenosine monophosphate (cAMP) accumulation assay and PCSK9 binding in an epitope competition assay (Fig. 1b,c and Supplementary Table 3). All but one of the 28 constructs expressed sufficiently for initial testing apart from the heavy chain GGSA linker fused with anti-PCSK9 Ab#1. Fusions with the [G_4_S]_3_A linker to the antibody light chain displayed potent GLP-1R activation (EC_50_: 100–300 pM) whilst PCSK9 binding activity decreased by 2-70-fold relative to the parental anti-PCSK9 antibodies. Based on PCSK9 binding data and manufacturability characteristics (i.e. expression level, fragmentation and aggregation propensity) of the antibody partner, the peptide fusion to the antibody light chain of Ab#2 with the [G_4_S]_3_A linker, denoted Ab#2_GLP1, was selected for further characterization. This fusion displayed kinetic parameters for binding to human PCSK9 (Fig. 1d) and potency in promoting LDL-C uptake in HepG2 cells treated with recombinant PCSK9 (Fig. 1e) that are comparable to Ab#2. In addition, Ab#2_GLP1 retained similar human GLP-1R potency and maximum efficacy relative to the control GLP-1 analogue peptide Fc fusion molecule GLP-1-Fc(γ4) (Fig. 1f).

**Figure 2:**
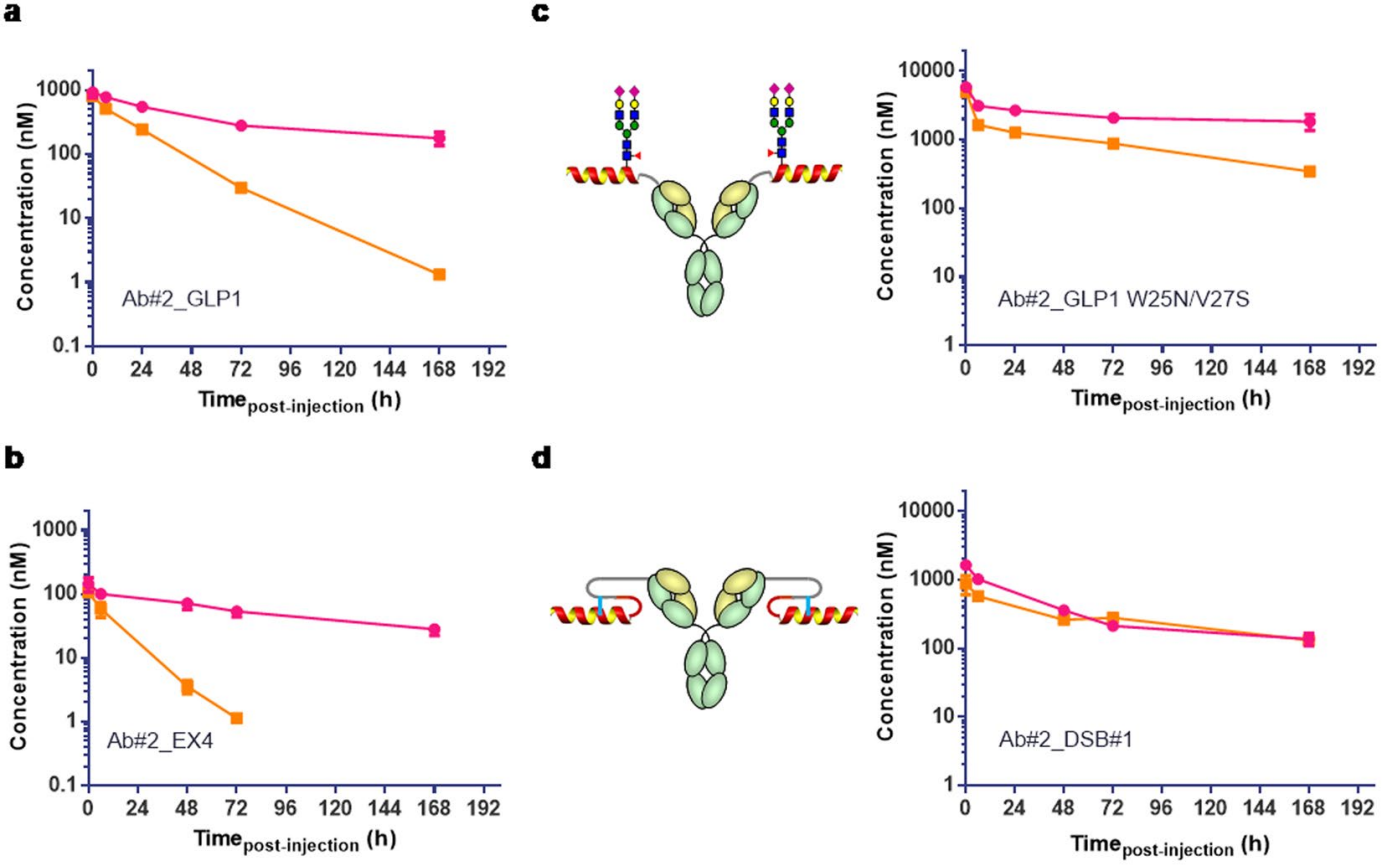
Stability engineering of GLP-1 analogue peptides in fusion with anti-PCSK9 Ab#2. Human Fc concentration (pink) and GLP-1R active compound concentration (orange) following single intravenous administration in C57BI/6 mice (n = 3 per time-point) of (**a**) Ab#2_GLP1 at 5 mg/kg; (**b**) Ab#2_EX4 at 1 mg/kg; (**c**) N-glycosylated GLP-1 analogue W25N/V27S Ab#2 fusion at 40 mg/kg; (**d**) disulphide bridge stabilised variant Ab#2_DSB#1 at 10.8 mg/kg. Values are presented as mean (±SEM). Concentration of the active compound at 168 h post-dose for Ab#2_EX4 was not reported as it was below the lower limit of quantification of the assay.

We next determined the *in vivo* circulating half-life of Ab#2_GLP1 in lean mice. After a single intravenous dose, the peptide-antibody fusion concentrations were quantified over time utilising capture and detection reagents specific to human Fc. As a surrogate for peptide integrity, the activity of the fusion molecule was assessed by its ability to activate human GLP-1R in an *ex vivo* bioassay. The terminal half-life of Ab#2_GLP1 was 82 h based on Fc detection and 19 h based on GLP-1R activity (Fig. 2a and Supplementary Table 4). This discordance between the antibody Fc and GLP-1R activity half-life suggested that the peptide was either inactivated or separated from the antibody moiety and rapidly cleared. To better understand the degradation of peptide-antibody fusion, the same GLP-1 analogue fused to an isotype control antibody was administered to rats and the fusion protein was recovered and analysed by mass spectrometry seven days post-dose. While the [G_4_S]_3_A linker between the peptide and antibody light chain remained intact (Supplementary Fig. 1a,b), the DPP-4 resistant GLP-1 analogue was cleaved after positions G2, S12 and W25 (Supplementary Fig. 1c,d). Such proteolytic cleavage in the peptide would lead to the accumulation of long half-life metabolites able to suppress PCSK9 but inactive against GLP-1R. This difference in GLP-1R and PCSK9 activity over time would result in suboptimal pharmacology for a dual activity fusion molecule. Hence, the stability of the GLP-1R agonist peptide component required improvement.

### Engineering peptide stability to generate sustained ***in vivo*** GLP-1R activation

Cleavage after position W25 was also detected during production for some of our initial GLP-1 analogue peptide-antibody fusions and we hypothesized that W25 would be particularly labile. However, mutating the peptide at position W25 when in light chain fusion with the anti-PCSK9 Ab#2 resulted in peptide truncation during production except for the W25K variant. Unfortunately this mutation provided no additional *in vivo* stability to the fusion molecule (Supplementary Fig. 2). Exendin-4 is a GLP-1 analogue derived from the salivary glands of the Gila monster lizard^13^. It encompasses a long C-terminal extension that forms a compact fold around W25 (termed tryptophan cage) reducing its solvent exposure^14^. This structure has been shown to improve peptide chemical stability and to extend *in vivo* activity^15,16^ and could provide some stability benefit when in antibody fusion. However, we found that the exendin-4 antibody light chain fusion (Ab#2_EX4) had a GLP-1R activity half-life of 11 h, with no improvement seen in stability over the Ab#2_GLP1 fusion molecule (Fig. 2b and Supplementary Table 4).

Taking a different approach, we then engineered the fusion molecule to create steric hindrance around the peptide and block its degradation by proteases either by creating an N-linked glycosylation site at position 25 of the GLP-1 analogue peptide (Ab#2_GLP1 W25N/V27S) or by introducing a disulphide bridge between the exendin-4 peptide and the linker (Ab#2_DSB#1).

The glycosylated fusion molecule Ab#2_GLP1 W25N/V27S was highly stable *in vivo*, exhibiting a GLP-1R activity terminal half-life of 77 h in mice (Fig. 2c and Supplementary Table 4). This compound presented a 350-fold reduction in potency at human GLP-1R, using the cAMP accumulation assay, to 30 nM as compared to 87 pM for the non-glycosylated Ab#2_GLP1 fusion. To detect GLP-1R activity in the *ex-vivo* assay over a sufficient period of time for accurate half-life determination, the dose of the glycosylated variant in the PK stability experiment had to be increased to 40 mg/kg compared to 5 mg/kg for the non-glycosylated fusion. More generally, doses of peptide-antibody fusions for testing *in vivo* stability of the GLP-1 activity were adjusted based on their human GLP-1R potency and material availability.

**Figure 3:**
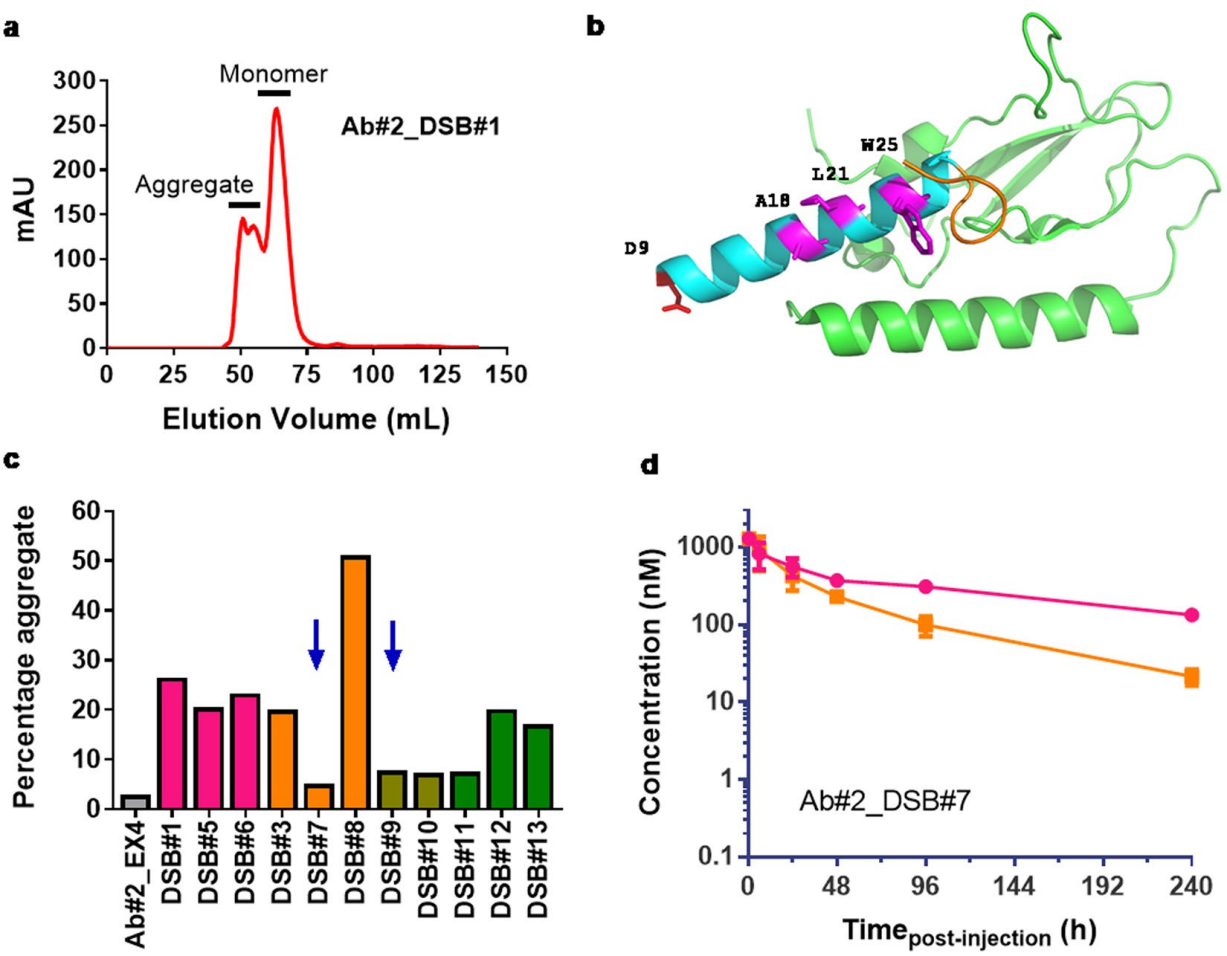
Optimising aggregation profile of disulphide bridge exendin-4 in anti-PCSK9 fusion. (**a**) Chromatogram profile of preparative size exclusion for Ab#2_DSB#1 compound following protein-A purification. (**b**) Structure (PBD 3C5T) of exendin-4 (cyan for the α-helix and orange for the tryptophan cage) in complex with the N-terminal domain of human GLP-1R (green) showing the targeted residues (pink) for rational design of peptide antibody fusion with low aggregation propensity. (**c**) Percentage aggregate in Protein-A purified samples of disulphide bridge variants in fusion with Ab#2 compared to exendin-4 fusion (Ab#2_EX4) − n = 1. Colour code corresponds to the cysteine mutation in the α-helix of exendin-4: D9C (pink), A18C (orange), L21C (khaki) and W25C (green). Fusions chosen for further analysis are shown with a blue arrow. (**d**) Human Fc concentration (pink) and GLP-1R active compound concentration (orange) following single intravenous administration in CD rats (n = 3) of Ab#2_DSB#7 at 10 mg/kg. For (**d**), values are presented as mean (±SEM).

In contrast, the potency at human GLP-1R of the exendin-4 disulphide stabilised fusion Ab#2_DSB#1 was better than the glycosylated variant Ab#2_GLP1 W25N/V27S although reduced compared to the non-modified exendin-4 antibody fusion (5.3 nM and to 110 pM, respectively). In addition, Ab#2_DSB#1 displayed equivalent terminal half-life in mice based on Fc detection and GLP-1R activity (Supplementary Table 4) with no significant loss of peptide activity for at least 7 days (Fig. 2d). Unfortunately, this fusion was highly prone to aggregation, making this construct a poor candidate for further development (Fig. 3a).

However, the concept of improving the peptide component stability through the introduction of a disulphide bridge appeared promising. The structure of exendin-4 in complex with the N-terminus domain of the human GLP-1R^17^ (Protein Data Bank code 3C5T) was then used to rationally guide the location of the cysteines in the α-helix of the peptide and in the linker or peptide C-terminus to minimize the impact on receptor binding and to favour disulphide bridge formation in order to reduce propensity to aggregate (Fig. 3b). Ten additional disulphide stabilised exendin-4 antibody fusions were produced (Supplementary Table 5) and, following protein A purification, four of these fusions presented a reduction in aggregate formation compared to Ab#2_DSB#1 (Fig. 3c). Two fusions, Ab#2_DSB#7 and Ab#2_DSB#9, were tested for GLP-1R activity and *in vivo* stability. DSB#7 and DSB#9 fusions displayed human GLP-1R potencies of 950 pM and 120 pM, respectively, which corresponds to an approximate 5 and 40-fold enhancement relative to the DSB#1 fusion. Following single intravenous administration in rats, the Ab#2_DSB#9 fusion did not show improvement in *in vivo* peptide stability with a GLP-1R activity half-life of 12 h. However, Ab#2_DSB#7 displayed a GLP-1R activity terminal half-life of 58 h (Fig. 3d and Supplementary Table 4), demonstrating improved *in vivo* peptide stability compared to unmodified GLP-1 analogue and exendin-4 fusions. This molecule with prolonged peptide activity half-life and low aggregation propensity formed the basis for further pharmacological optimisation.

### Engineering peptide-antibody fusion molecules with reduced GLP-1R potency

Dose dependent gastrointestinal adverse events are well-known effects associated with GLP-1 therapy^18^. To limit such effects at the high doses and exposure levels associated with effective PCSK9 neutralisation, the potency of the GLP-1 peptide component in the fusion was optimised. For this, a mechanism-based PK-PD *in silico* model was first developed to inform on an appropriate human GLP-1R potency. This algorithm simultaneously models the exposure-response relationship between the fusion molecule, the soluble target PCSK9 and GLP-1R activity based on potency in the cAMP assay. Simulations predicted that for a compound with a human PCSK9 affinity below 1 nM, a once-weekly 50 mg dose would achieve robust free PCSK9 suppression (Supplementary Fig. 3a). By testing a theoretical range of human GLP-1R potencies at a once-weekly 50 mg dose and assuming the peptide retains its activity over the dosing period, simulations showed that a potency 40-fold less than the reference GLP-1-Fc(γ4) molecule should deliver equivalent GLP-1 activity to a 1.5 mg weekly dose of GLP-1-Fc(γ4) (Supplementary Fig. 3b).

**Figure 4:**
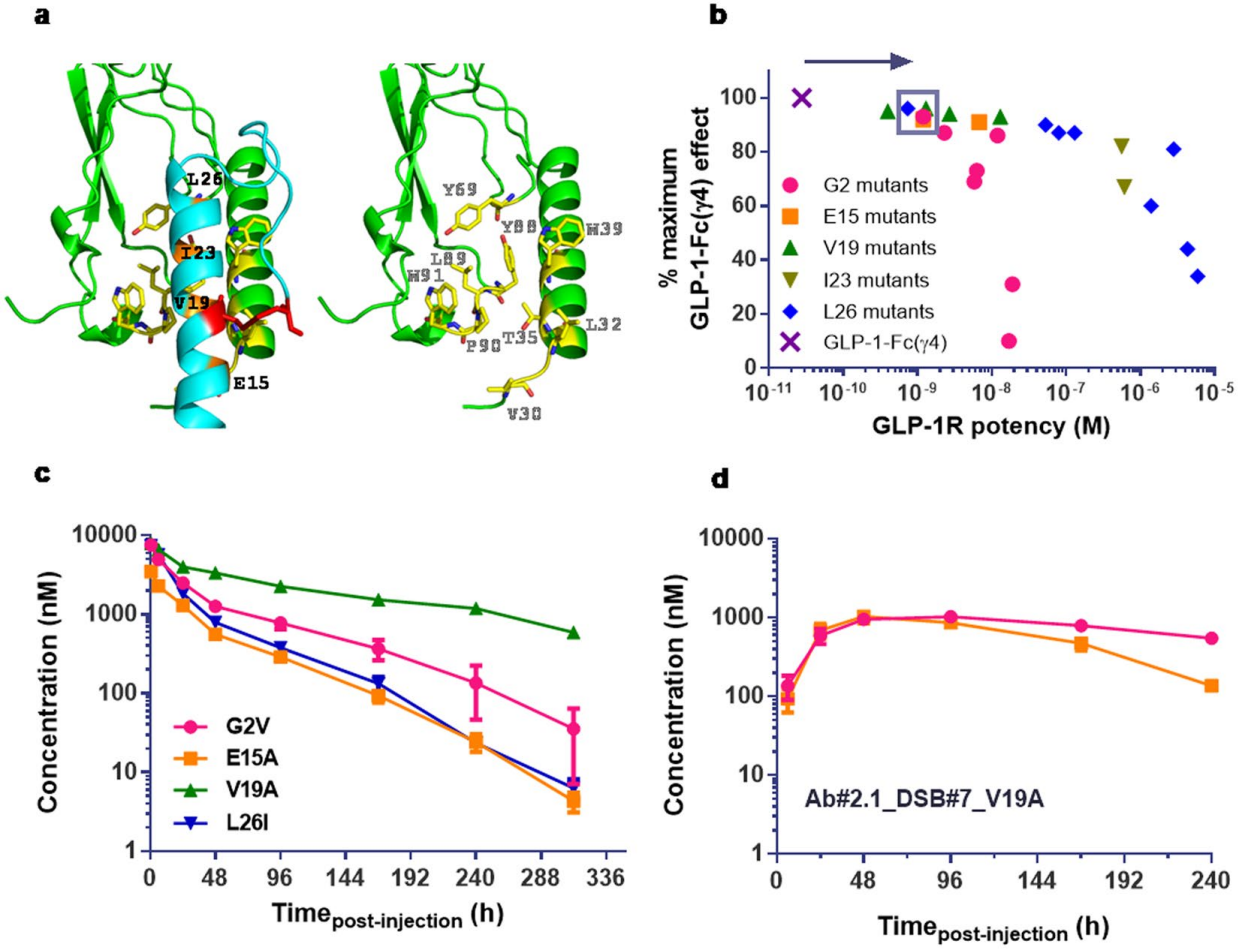
Optimising GLP-1R potency of disulphide bridge DSB#7 peptide in antibody fusion. (**a**) Split view model of DSB#7 analogue peptide (cyan) in complex with the N-terminal domain of human GLP-1R (green) showing in orange the targeted residues for rational design of peptide antibody fusion with reduced potency at human GLP-1R. Receptor residues predicted to interact with the targeted peptide amino acids are shown in yellow. The disulphide bridge between the peptide α-helix and C-terminus is shown in red. (**b**) Activity at human GLP-1R expressed in CHO cells using the cAMP accumulation assay for DSB#7 peptide variants with single point amino acid mutation at G2 (pink), E15 (orange), V19 (green), I23 (khaki) and L26 (blue) in fusion with the optimised antibody Ab#2.1. Reference compound GLP-1-Fc(γ4) is shown as a purple cross. Values are shown as mean of n = 2. Fusion molecules at target human GLP-1R potency are shown in a grey square. (**c**) Human Fc concentration following single intravenous administration in CD rats (n = 3 per compound) of Ab#2.1 in fusion with DSB#7 peptide variant G2V (pink) at 60 mg/kg, E15A (orange) at 53 mg/kg, V19A (green) at 58.5 mg/kg and L26I (blue) at 60 mg/kg. (**d**) Human Fc concentration (pink) and GLP−1R active compound concentration (orange) following single subcutaneous administration at 60 mg/kg in CD rats (n = 3) of the fusion Ab#2.1_DSB#7_V19A. For (**c**) and (**d**), values are presented as mean (±SEM).

The anti-PCSK9 antibody Ab#2 was affinity matured by rational design using a structure homology model of the mAb in complex with human PCSK9 to generate the optimised version Ab#2.1, which should deliver robust PCSK9 suppression at lower doses than Ab#2. The disulphide bridge stabilised DSB#7 peptide fused to Ab#2.1 was only 7-fold less potent than GLP-1-Fc(γ4) at human GLP-1R in the cAMP assay using Chinese hamster ovary (CHO) transfected cells, indicating that the potency needed to be further reduced (Supplementary Fig. 4). To rationally optimize peptide potency, a homology model of DSB#7 in complex with the GLP-1 receptor was generated (Fig. 4a). Positions predicted to be involved in binding to the receptor e.g. E15, V19, I23, L26 as well as G2, a residue critical for receptor activation^19^, were mutated and variants tested for human GLP-1R potency and maximum activation compared to GLP-1-Fc(γ4) in the cAMP accumulation assay. Potency of these variant fusions ranged over several orders of magnitude and some of the mutations at positions G2, L26 and I23 resulted in partial agonism (Fig. 4b and Supplementary Table 6). However, four fusions with a single point mutation (G2V, E15A, V19A and L26I) had potency at human GLP-1R similar to the target value and were investigated further for *in vivo* peptide stability and manufacturability.

### Identification of the peptide-antibody fusion MEDI4166

Following expression, V19A and L26I variants showed the highest percentage of monomeric product post-protein A purification and the lowest aggregation after a 4 week 5 °C incubation at the target concentration of 50 mg/mL (Supplementary Fig. 5). Following single intravenous administration in rats, the V19A variant molecule had the best pharmacokinetic profile out of the four fusions (Fig. 4c), equivalent to the anti-PCSK9 antibody Ab#2.1 (Supplementary Fig. 6 and Supplementary Table 4). The V19A fusion was also highly stable *in vivo* with no loss of GLP-1R activity compared to Fc exposure for up to 4 days following single subcutaneous administration in rats (Fig. 4d). This peptide-antibody fusion molecule, Ab#2.1_DSB#7_V19A, was called MEDI4166. It exhibited an attractive manufacturability profile notably due to its low aggregation propensity during production (Supplementary Fig. 7). The molecule has the same affinity for human PCSK9 as Ab#2.1 and cross-reacted with cynomolgus and rat PCSK9 (Supplementary Table 7). MEDI4166 neutralised PCSK9 binding to the LDL receptor and restored LDL-C uptake in HepG2 cells (Supplementary Fig. 8). In addition, potency of MEDI4166 for human GLP-1R in transfected CHO cells displayed a 45-fold reduction compared to the GLP-1-Fc(γ4) control molecule in the cAMP accumulation assay (Supplementary Table 8). The compound is highly specific for GLP-1R, as it is unable to activate the closely related human receptors for glucagon, glucagon-like peptide-2 (GLP-2) and secretin. A small activation of the gastric inhibitory polypeptide (GIP) receptor was detected at concentrations above 1 µM (Supplementary Fig. 9). Importantly, MEDI4166 was able to induce glucose-stimulated insulin secretion in INS1 β-cells expressing endogenous GLP-1R (Supplementary Fig. 10) with a reduced potency compared to GLP-1(7–36) amide peptide (147 nM and 1.2 nM respectively). In this assay, maximum insulin level for MEDI4166 was 65% of the reference peptide. To conclude, MEDI4166 has been engineered to be a novel long acting GLP-1R agonist peptide with a reduced potency in fusion with an optimized neutralising anti-PCSK9 antibody to deliver glycaemic and weight control in combination with LDL-C lowering.

### MEDI4166 demonstrates extended glucose control, delays development of diabetes and reduces obesity in rodent models

**Figure 5:**
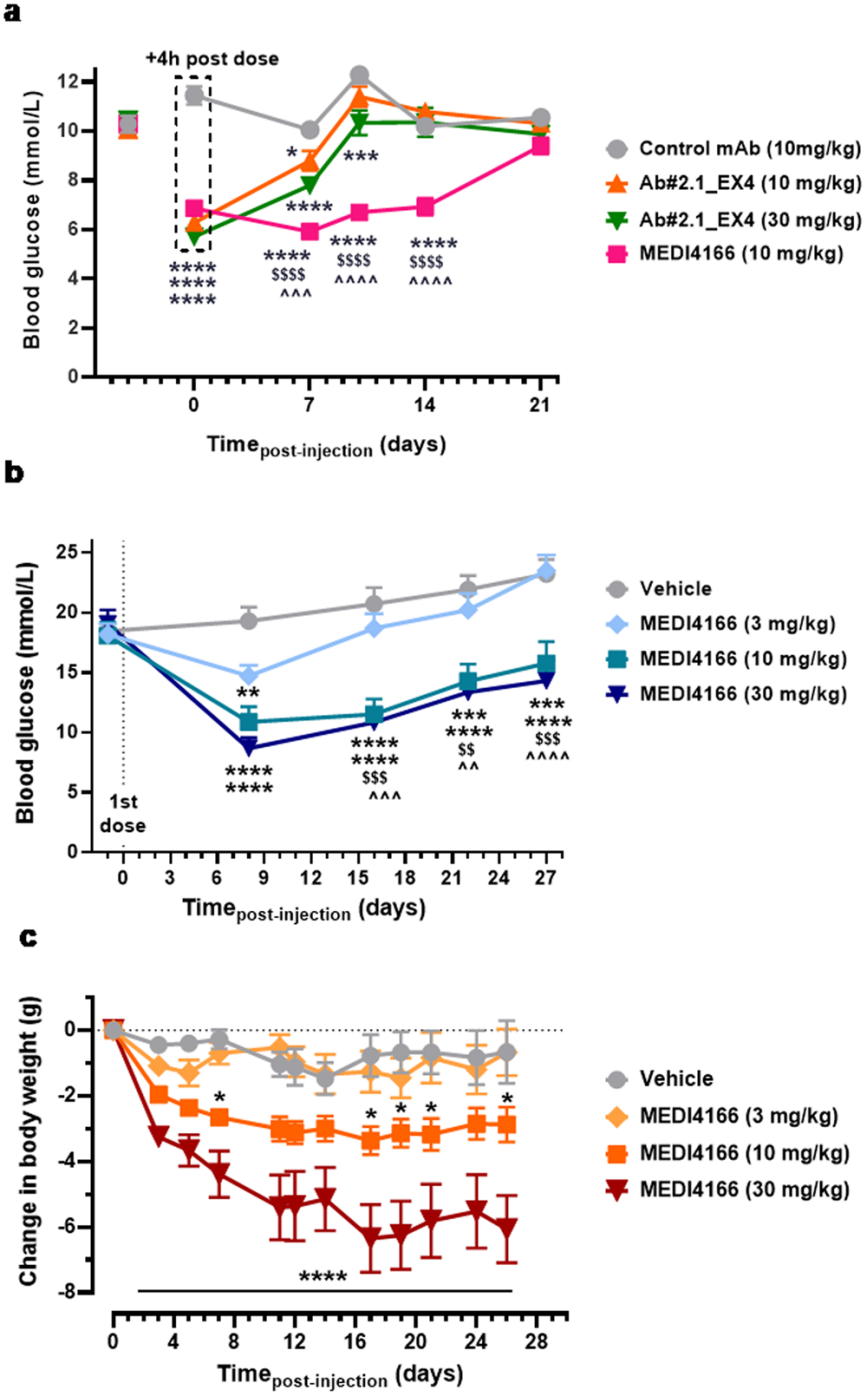
Glucose control and weight loss induced by MEDI4166 in mouse models of diabetes and obesity. (**a**) Fasted blood glucose levels in male DIO mice (n = 10/group) following a single subcutaneous administration of anti-PCSK9 control antibody Ab#2.1 (10 mg/kg), MEDI4166 (10 mg/kg) or Ab#2.1_EX4 (10 or 30 mg/kg). Values are presented as mean (±SEM). In all cases, *p < 0.05; ***p < 0.001; ****p < 0.0001 compared to control mAb; ^$$^p < 0.01; ^$$$$^p < 0.0001 MEDI4166 compared to 10 mg/kg Ab#2.1_EX4; ^p < 0.05; ^^^p < 0.001; ^^^^p < 0.0001 MEDI4166 compared to 30 mg/kg Ab#2.1_EX4; ^##^p < 0.01 30 mg/kg Ab#2.1_EX4 compared to 10 mg/kg Ab#2.1_EX4. (**b**) Fasted blood glucose levels in male diabetic *db/db* mice (n = 12/group) following weekly subcutaneous administration of vehicle (PBS) or MEDI4166 (3, 10 or 30 mg/kg) for 4 weeks. Values are presented as mean (±SEM);. **p < 0.05; ***p < 0.001; ****p < 0.0001 compared to vehicle. ^$^p < 0.05; ^$$^p < 0.01; ^$$$^p < 0.001 3 mg/kg MEDI4166 compared to 10 mg/kg MEDI4166. ^p < 0.05; ^^p < 0.01; ^^^p < 0.001; ^^^^p < 0.0001 3 mg/kg MEDI4166 compared to 30 mg/kg MEDI4166. (**c**) Body weight change of DIO mice (n = 8/group) following once weekly subcutaneous administration of vehicle (PBS) or MEDI4166 (3, 10 or 30 mg/kg) for 26 days. Values are presented as mean ( ± SEM); ****p < 0.0001 for MEDI4166 (30 mg/kg) vs. vehicle; *p < 0.0001 for MEDI4166 (10 mg/kg) vs. vehicle.

To investigate if the optimised *in vivo* stability and reduced potency of the MEDI4166 GLP-1 peptide component translates into prolonged glucose control, we compared the impact of MEDI4166 and exendin-4 in fusion with the same antibody partner (Ab#2.1_EX4) on fasting plasma glucose (FPG) levels in moderately hyperglycaemic diet-induced obese (DIO) mice over 21 days (Fig. 5a). Following a single subcutaneous dose, both MEDI4166 and Ab#2.1_EX4 significantly reduced FPG 4 h post-dose. However, the glucose-lowering effects of MEDI4166 persisted for up to 14 days before returning to baseline levels on day 21 whereas the effects of Ab#2.1_EX4 approached baseline levels 10 days post-treatment. Glucose tolerance tests (GTT) were also performed at day 0 and day 10 to characterize duration of effect. At 4 h post dose (day 0), both molecules significantly reduced glucose excursion, but only MEDI4166 reduced glucose excursion at day 10 (Supplementary Fig. 11). Consistent with these effects, the GLP-1 activity of MEDI4166 in the *ex-vivo* cAMP accumulation assay was maintained out to 21 days in contrast to Ab#2.1_EX4 which approached baseline levels on day 7 (Supplementary Fig. 12). Together these data demonstrate that in DIO mice the GLP-1 peptide component of MEDI4166, despite being 52- and 37-fold less potent than Ab#2.1_EX4 at mouse GLP-1R in CHO transfected cells and MIN6 β-cells respectively (Supplementary Table 9), resulted in more durable glucose control than the non-optimised fusion. In addition, MEDI4166 has no impact on glucose excursion following a GTT in GLP-1R knock-out mice (Supplementary Fig. 13) demonstrating that its effects on glucose are mediated through specific engagement of the GLP-1R.

The ability of MEDI4166 to improve glycaemic control in a model of Type 2 diabetes was investigated in male *db/db* mice following weekly injections of MEDI4166 at 3, 10 or 30 mg/kg over a 4 weeks period. Glucose levels in 4 h-fasted animals were reduced in MEDI4166-treated mice in a dose-dependent manner with the mid and high doses significantly delaying the progression of diabetes (Fig. 5b). A GTT conducted on day 22 showed that both 10 and 30 mg/kg MEDI4166 treatment were able to significantly improve glucose tolerance (Supplementary Fig. 14).

The impact of MEDI4166 on body weight following repeated dosing was evaluated in DIO mice. Weekly injections of MEDI4166 reduced body weight in a dose-dependent manner (Fig. 5c) with the 30 mg/kg treatment delivering a 13% decrease in body weight compared to a 1.4% reduction for the control group at day 26. Body weight loss in the MEDI4166 30 mg/kg dose group was accompanied by a reduction in food intake (Supplementary Fig. 15) and fat mass, but not fat free mass (Supplementary Table 10). Analysis of clinical chemistry parameters measured at the end of the 4-week study (Supplementary Table 11) showed that MEDI4166 treatment reduced FPG, total cholesterol and serum alanine transaminase (ALT) levels, which is indicative of improved liver function.

### MEDI4166 efficiently suppresses PCSK9 and reduces LDL-cholesterol in cynomolgus monkey

**Figure 6:**
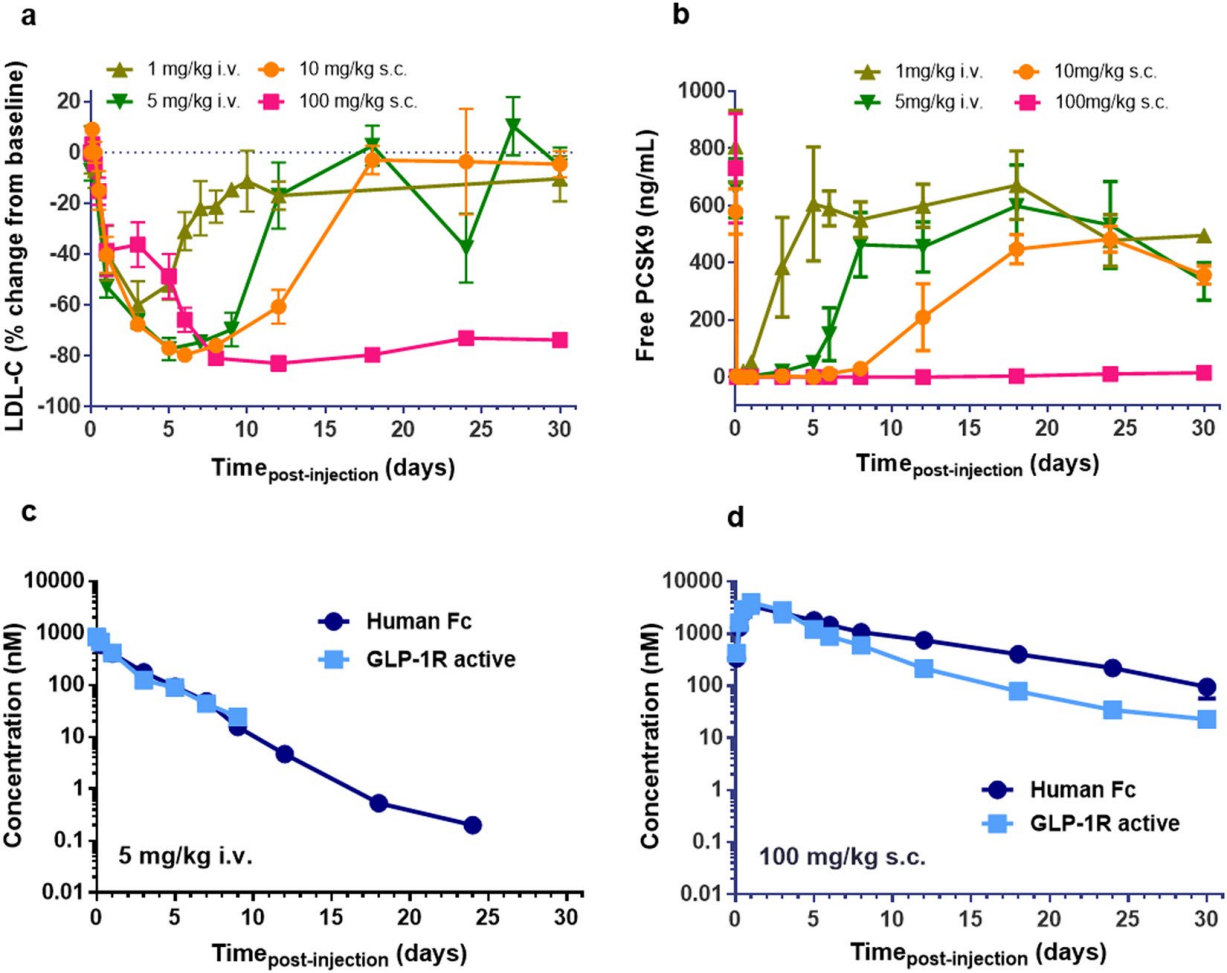
MEDI4166 anti-PCSK9 activity and exposure profiles in lean cynomolgus monkey following single injection. (**a**) Change in plasma LDL-C and (**b**) free PCSK9 concentration following MEDI4166 intravenous administration at 1 (khaki) or 5 (green) mg/kg or subcutaneous administration at 10 (orange) or 100 (pink) mg/kg. (**c**) Human Fc concentration (dark blue) and GLP-1R active compound concentration (light blue) following intravenous administration of MEDI4166 at 5 mg/kg or (**d**) subcutaneous administration at 100 mg/kg. n = 3 animals per group. Values are presented as mean (±SEM). Concentration of GLP-1R active compound after 9 days and Fc concentration after 24 days for MEDI4166 at the 5 mg/kg (iv) dose were not reported as levels were below the lower limit of quantification of the assays.

The effects of MEDI4166 on PCSK9 suppression, LDL-C level and the pharmacokinetic profile based on human GLP-1R activity and Fc detection, to determine *in vivo* peptide stability, were evaluated in healthy lean cynomolgus monkeys following a single intravenous (iv) dose at 1 or 5 mg/kg or subcutaneous (sc) dose at 10 or 100 mg/kg. No adverse effects were observed during the study and food consumption remained unaffected by treatment. A slight decline in bodyweight was observed for the first 6 days in the 100 mg/kg subcutaneous treated group (−7% compared to day 0 baseline at day 6) before increasing thereafter (Supplementary Fig. 16). MEDI4166 at 10 mg/kg (sc) decreased LDL-C up to 80% at day 6 before returning to baseline at day 18 (Fig. 6a). This data correlated well with the free PCSK9 level which was not efficiently suppressed after day 8 (Fig. 6b). Following treatment with the high dose (100 mg/kg, sc), PCSK9 levels were completely suppressed for at least 30 days leading to a LDL-C reduction of greater than 70% over the duration of the study. The pharmacokinetic profiles for Fc and human GLP-1R active compound following administration at 5 mg/kg (iv) and 100 mg/kg (sc) are shown in Fig. 6c and d respectively. MEDI4166 is fully stable in cynomolgus monkey for at least 9 days following intravenous administration with no discordance between Fc and GLP-1R active compound concentrations. MEDI4166 is also highly stable following subcutaneous administration with no more than 2-fold difference between Fc and active compound concentrations for at least 8 days. The terminal half-life of MEDI4166 in cynomolgus monkeys based on Fc and GLP-1R activity in the 100 mg/kg (sc) group were determined to 147 and 116 h respectively (Supplementary Table 4).

## Discussion

Peptides and antibodies are two major classes of drug with impressive clinical and commercial credentials^20,21^. However, to the best of our knowledge, no dual activity peptide-antibody fusion molecule has been tested in the clinic so far. Considering the rapid progress of bispecific antibody based molecules^22^, this lack of clinical data for any peptide-antibody fusion likely reflects the significant technical challenges in the development of such molecules. Indeed, generating a fusion or conjugation between a peptide and a carrier molecule such as an antibody Fc fragment^23,24^ or a full antibody^25,26^ is a well described half-life extension strategy to address their rapid clearance from circulation and improve their therapeutic potential. However, there are only a few reports of therapeutic peptides in fusion with a functional antibody to deliver multiple activities in a single molecule. Examples of such fusions are Ang2 blocking peptides fused to the anti-TNF adalimumab^27^ or the anti-ErbB2 trastuzumab antibodies^28^ as well as an IL-17A neutralising peptide fused to an anti-IL-22 antagonistic antibody^29^. For the IL-17A/IL-22 fusion, significant protein engineering was required to obtain a dual activity profile in *in vitro* assays and to limit the peptide degradation during production, a phenomenon frequently observed for peptide fusion molecules expressed in transfected cells^30^. In addition, despite addressing the degradation on production, the peptide component of this IL17A/IL-22 fusion was still prone to cleavage after *in vivo* administration^31^. *In vivo* degradation of linear Ang2 binding peptide was also detected when in fusion with trastuzumab^28^. Such relative instability of the peptide *in vivo*, compared to the antibody component, will lead to suboptimal efficacy of the fusion molecule. Therefore, generating a peptide-antibody fusion with sustained activity for both the antibody and the peptide driven pharmacology is a challenge that is essential to overcome to maximise the therapeutic benefits of those fusion molecules.

Here we report the development of MEDI4166; a long acting GLP-1 analogue peptide fused to an anti-PCSK9 antibody. The peptide component of MEDI4166 has been extensively optimised to provide lasting glucose and weight control that is able to better match the antibody PK in combination with a reduced GLP-1R potency and a favourable manufacturability profile.

The initial step in designing a PCSK9/GLP-1 dual molecule was to fuse a DPP-4-resistant GLP-1 analogue peptide with either GGSA or [G_4_S]_3_A linkers to the heavy or light chain variable domains of a panel of anti-PCSK9 antibodies. Activities of the fusions were highly variable, ranging from 74 pM to 33 nM potency at human GLP-1R and up to 120-fold loss of PCSK9 binding compared to the parent antibodies (Fig. 1b,c). Impact of the fusion on PCSK9 binding is likely due to steric clashes generated by the peptide moiety. For instance, structural analysis of the anti-PCSK9 mAb 1D05^32^ reveals that the light chain N-terminus is close to the binding interface and a peptide fusion at this locus is likely to significantly alter the antibody binding properties. Differences in GLP-1R potencies observed in the panel of fusion constructs may also be explained by varying degrees of steric hindrance. Whilst we did not investigate the reasons for each potency variation, it is also possible that a fraction of the peptide component may self-associate or bind to the antibody surface thus reducing its ability to effectively engage with the receptor. Indeed, GLP-1 and analogues are known to form amphipathic α-helix structures that are poorly soluble and prone to generate aggregates^33^.

The PK stability profile in mice of our early fusions such as Ab#2_GLP1 and Ab#2_EX4 demonstrated that the *in vivo* GLP-1R activity of such peptide-antibody fusion molecules rapidly decreased over time due to peptide degradation compared to the exposure achieved by the antibody Fc component (Fig. 2a and b). The activity half-life in rat of exendin-4 in fusion with a carrier antibody has been previously reported as 10.8 h^26^, which is similar to the 11.5 h value we observed in mice for exendin-4 in fusion with the anti-PCSK9 Ab#2 antibody (Supplementary Table 4). Numerous proteolytic enzymes are involved in the GLP-1 peptide degradation pathways^34^ and eliminating all possible cleavage sites via amino acid mutation, without dramatically impacting on GLP-1R activity, would be particularly challenging, or indeed may not be possible. For instance, mutating the W25 peptide amino acid, a residue we identified as being particularly labile, led to truncation during production or to no *in vivo* stability benefit (Supplementary Fig. 2). We therefore investigated a more generic approach consisting of generating steric blockade around the peptide to render it protease-resistant. This was investigated by either creating a disulphide bridge between the peptide and the linker or by adding an N-linked carbohydrate moiety.

Glycoengineering has been successfully used to increase exposure and pharmacodynamics of darbepoetin, a hyperglycosylated analogue of erythropoietin^35^ and our glycosylated GLP-1 analogue peptide fusion variant Ab#2_GLP1 W25N V27S showed indeed *in vivo* stability improvement (Fig. 2c). Unfortunately, this compound has a too low GLP-1R potency to offer a viable therapeutic solution. As an alternative approach to improve *in vivo* stability of the fusion molecule, we then engineered a disulphide bridge into the exendin-4 peptide moiety (Ab#2_DSB#1) as this has been shown to improve *in vitro* and *in vivo* stability of both GLP-1 and exendin-4 analogue peptides^36^. Ab#2_DBS#1 has a reduced GLP-1R potency compared to the parent fusion but to a lesser extent than the glycosylated Ab#2_GLP1 W25N V27S (5.3 and 30 nM respectively). Remarkably, Ab#2_DSB#1 retained full GLP-1R activity for up to 7 days in mice with a terminal half-life of 83 h (Fig. 2d) which we believe is, excluding slow release formulation technology, the longest half-life for any GLP-1 analogue described to date. Ultimately, the optimized lead molecule MEDI4166 displayed robust glucose-lowering effects that persisted for up to 14 days following a single dose in DIO mice, whereas blood glucose levels approached baseline levels for the non-stabilized control molecule (Fig. 5a). In the same mouse model, MEDI4166 also demonstrated efficient glucose control following a GTT performed 10 days after a single administration (Supplementary Fig. 11). The prolonged GLP-1R activity of MEDI4166 can also be observed, using the *ex-vivo* cAMP accumulation assay, in rat (Fig. 4d) and cynomolgus monkeys (Fig. 6c,d) demonstrating that the peptide stability improvement, conferred by the disulphide bridge, is translatable across species and independent of the different protease environments.

Introducing steric blockade has proven a successful strategy to increase the stability of a peptide *in vivo* but typically this has been at the expense of potency, as for instance a 48-fold difference at human GLP-1R in transfected CHO cells between Ab#2_DSB#1 and the same molecule without the disulphide bridge (Ab#2_EX4). From a therapeutic point of view, it should be possible to overcome the lower potency of the compound by using higher doses to achieve the desired outcomes. However, the benefits expected of an improved stability molecule with a lower potency need to be carefully weighted against other considerations such as manufacturing and formulation requirements as well as patient convenience and compliance.

Since the two different pharmacological activities of the peptide antibody fusion molecule cannot be titrated separately, precise adjustment of the potency at GLP-1R was required to achieve effective and safe GLP-1 activity at doses likely able to suppress PCSK9. As clinical data for both anti-PCSK9 antibodies and GLP-1 analogue molecules are widely available, we used PK-PD modelling to determine the appropriate potency at human GLP-1R for the fusion molecule that should deliver robust glucose control without inducing non-manageable GLP-1 driven adverse events at exposure levels able to efficiently neutralize PCSK9. It is well described that relatively high doses of anti-PCSK9 antibodies, as for instance 140 mg every two weeks of evolocumab, are required to overcome the antigen sink^37,38^. The direct consequence of such a high dose is that the potency of the GLP-1 peptide component in the fusion had to be appropriately reduced to try minimising GLP-1-induced gastrointestinal side effects^18^. Indeed, even with a 1.5 mg weekly dose of the GLP-1 analogue Fc fusion drug dulaglutide, approximately 20% of patients experienced nausea in the AWARD-3 clinical trial^39^. Reducing potency of the GLP-1 analogue peptide may appear counterintuitive for a potential therapeutic but in this case, it has enabled us to incorporate significant stability improvement in the MEDI4166 molecule.

In addition to demonstrating *in vitro* cAMP activity at human and mouse GLP-1R in transfected CHO and in the mouse MIN6 insulinoma cells, MEDI4166 induced glucose-stimulated insulin secretion in INS1 β-cells and displayed a desirable profile in rodent disease models. It reduced fasting glucose and improved glucose tolerance in both DIO and *db/db* mice as well as inducing weight loss in DIO mice. In lean cynomolgus monkeys, MEDI4166 demonstrated robust PCSK9 suppression and LDL-C reduction. The duration of action and maximum LDL-C reduction in non-human primates, around 80% change from baseline for MEDI4166, are broadly comparable to others anti-PCSK9 mAbs^32,40,41^. Time for returning to baseline LDL-C level might be slightly shorter using MEDI4166, around 10 and 14 days following a 1 mg/kg iv dose of MEDI4166 and anti-PCSK9 J16 mAb^42^ respectively. If confirmed, this could eventually be due to small differences in bioavailability, distribution or elimination of the peptide-antibody fusion compared to conventional mAbs.

To conclude, our data support further investigations of MEDI4166 as a potential treatment for T2D patients with high cardiovascular risk. However, as is often the case for many new medicines, there could be limitations in translating pre-clinical effects to the patient population. For instance, LDL-C decrease observed in lean cynomolgus monkeys might not accurately predict efficacy in patients with severe hypercholesterolemia. Obese and diabetic non-human primates^43,44^ could be very helpful animal models to analyse the pharmacology of MEDI4166 for both glucose control and LDL-C reduction and might provide additional insights to precisely predict its efficacy in humans.

Bispecific molecules are more often designed to target antigens expressed on the same cell or cells within the same environment but our data also demonstrates the feasibility of achieving dual pharmacology when targeting molecules in very different compartments: a soluble serum protein (PCSK9) and a GPCR predominately expressed in pancreatic β-cells (GLP-1R). GLP-1R expression has also been demonstrated in the central nervous system^45^ (CNS), but it is unlikely that such a large molecule as MEDI4166 can efficiently cross the blood brain barrier. In addition, we have shared a broadly applicable methodology for the engineering of peptide-antibody fusion molecules that enables long *in vivo* stability and activity whilst balancing dual functionality and improving its manufacturability profile. Peptides and antibodies represent two important therapeutic modalities and combining them in a single entity affords the ability to incorporate diverse mechanism of actions such as antagonistic and agonistic activities in distinct compartments as exemplified here. Versatility and flexibility of the peptide-antibody fusion format could be particularly well suited to address complex diseases by targeting several biological pathways and offer new therapeutic approaches.

## Methods

### Peptide-antibody fusion constructs

The genes encoding the light and heavy chain variable domains of the anti-PCSK9 antibodies were synthesized by GeneArt, amplified by PCR and cloned into the mammalian expression vectors previously described^46^. DNA coding for the DPP-4 resistant GLP-1 analogue peptide in fusion with the [G_4_S]_3_A or GGSA amino acid linker, as well as for exendin-4 peptide in fusion with the [G_4_S]_3_A linker, were also synthesized by GeneArt. The different fusion genes were then assembled by overlapping PCR before cloning into the mammalian expression vectors. Site directed and saturation mutagenesis were performed using the Quikchange II kit (Agilent Technologies) or by overlapping PCR. All constructs were confirmed by DNA sequencing before transfection.

### Peptide-antibody fusion production

Anti-PCSK9 antibodies and peptide-antibody fusions were transiently expressed by co-transfection of the heavy and light chain expressing vectors into CHO cells using a polyethylenimine based method^47^. Supernatants were purified by affinity chromatography using a Protein A MabSelectSure column (GE Healthcare) and compounds were analysed for aggregation and degradation by SDS-PAGE and SEC-HPLC using a TSKgel G3000SWxl column (Tosoh Bioscience). Compounds used for *in vivo* experiments were further purified by size exclusion chromatography using a HiLoad Superdex 200 pg column (GE Healthcare) and further characterised for endotoxin level using the LAL Kinetic-QCL (Lonza) and integrity by LC/MS using an Acquity UPLC system coupled to a Synapt G1 mass spectrometer (Waters).

### Cell lines

Stable CHO cell lines expressing human and mouse GLP-1R were generated in-house using standard methods as previously described^48^. Human GLP-1R CHO cells were cultured in Dulbecco’s modified Eagle’s medium (DMEM) supplemented with 10% foetal bovine serum (FBS), 0.5 mg/mL geneticin and 0.4 mg/mL hygromycin. Mouse GLP-1R CHO cells were cultured in Hams F12/DMEM (1:1) with L-glutamine (Invitrogen) supplemented with 10% FBS and 0.8 mg/mL geneticin. MIN6 mouse insulinoma cells were licenced from University of Osaka to AstraZeneca and were cultured in DMEM supplemented with 10% FBS and 50 µM 2-mercaptoethanol. Cells were frozen as 1 mL aliquots containing 1–1.5 × 10^7^ cells and separate aliquots were used for individual cAMP accumulation experiments. HepG2 cells were purchased from ECACC and were cultured in Minimum Essential Medium supplemented with 10% FBS and 1% non-essential amino acids (Invitrogen).

### Cyclic AMP accumulation assay

Potency of peptide-antibody fusions at the GLP-1 receptor was assessed using a cAMP accumulation assay. CHO cells expressing human GLP-1R were dispensed at 1 × 10^5^ cells/mL in assay buffer (Hanks Balanced Salt Solution containing 0.1% BSA (Sigma-Aldrich) and 0.5 mM IBMX (Sigma-Aldrich)) in 384-well assay plates (5 µL per well) before adding dilutions of tested compounds prepared by a manual tip-based method. After 30 min incubation, cAMP levels were measured using the cAMP dynamic 2 HTRF® kit (Cisbio) following manufacturer’s recommendations. Fluorescence emissions at 665 nm and 620 nm following excitation at 320 nm were detected using an Envision reader (Perkin Elmer) and data were transformed to % Delta F as described in manufacturer’s guidelines. Compound potency in CHO cells expressing mouse GLP-1R or MIN6 cells were determined as reported previously^48^ using a non-contact acoustic dispensing method for compound dilution.

### PCSK9 binding assay

Binding of peptide-antibody fusions to human PCSK9 were compared to their respective anti-PCSK9 antibody partners using a HTRF® epitope competition assay. Each anti-PCSK9 mAb was labelled with DyLight-650 fluorophore (Thermo Fisher Scientific). Recombinant human PCSK9 with an N-terminal Avi-tag (in-house) was biotinylated using the biotin ligase BirA (Avidity LLC) as per the manufacturer’s instructions. Titration of peptide-antibody fusions or unlabelled anti-PCSK9 mAbs were prepared in assay buffer (1X PBS, 0.1% BSA and 0.4 M Potassium Fluoride) and transferred to 384-well assay plates. Samples were incubated for 4 h with 1 nM streptavidin cryptate (Cisbio), 0.05-0.5 nM of biotinylated PCSK9 and 0.1–2 nM of labelled anti-PCSK9 antibody. Fluorescence was read out as described above and results were expressed as percent of specific binding.

### LDL-cholesterol uptake by HepG2 hepatic cells

Ability of anti-PCSK9 antibodies and peptide-antibody fusions to block PCSK9 activity and restore LDL-C uptake was tested *in vitro* by plating in 96-well plates 2 × 10^4^ HepG2 cells per well in DMEM medium supplemented with 10% lipoprotein deficient serum and cultured overnight. Tested compounds were pre-incubated for 1 h with 45 nM of human PCSK9 before being transferred to the cell plates. After 1 h, 50 nM of Bodipy LDL-C (Molecular Probes) was next added to the cells followed by a 5 h incubation. Cells were washed thoroughly with PBS, stained with the nuclear dye Hoescht and fixed using 3.7% formaldehyde. Assay plates were read for cell-associated fluorescence using a Cellomics ArrayScan VTi (Thermo Scientific) high content imaging system.

### Animal studies

All rodent studies were approved either by the Göteborg Preclinical Ethical Committee for AstraZeneca (Sweden), the Institutional Animal Care and Use Committee at MedImmune (Gaithersburg, MD, USA) in accordance with US Animal Welfare Act guidelines, Home Office licences for studies performed at MedImmune (Cambridge, UK), or under personal licenses issued by the Danish Committee for Animal Research for studies performed at Gubra (Horsholm, Denmark).

Cynomolgus monkeys were obtained by a certified provider and the study was subjected to the provisions of the United Kingdom Animals Act 1986 and under UK Home Office licence.

### Single-dose study in diet-induced obese (DIO) mice

Male C57Bl/6 mice (Jackson Labs; 20–21 weeks of age), maintained on a 60% kcal/fat diet (Research Diets) for 15 weeks, were singly housed upon arrival and allowed to acclimatize for 3 weeks. Mice were randomized to one of the four treatment groups (n = 10/group) based on body weight and 6 h-fasted glucose measured on day -4: control antibody (10 mg/kg), MEDI4166 (10 mg/kg) or Ab#2.1_EX4 (10 or 30 mg/kg). Test compounds were administered subcutaneously following a 2 h fast on day 0. Fasting glucose measurements were recorded 4 h post-injection (day 0) as well as on day 7, 10, 14, and 21. An intraperitoneal glucose tolerance test (IPGTT) was performed on days 0 and 10. Additional mice (n = 3/group) were administered with MEDI4166 at 10 mg/kg or Ab#2.1_EX4 at 10 or 30 mg/kg to determine human Fc and GLP-1R active compound concentrations by taking blood samples at the following times post-dosing: 2 h, 4 h, 8 h, 1, 3, 7, 14 and 21 days.

### Repeat-dose study in diet-induced obese mice

Male DIO mice, maintained on a 60% kcal/fat diet for 15 weeks, were switched to 45% kcal/fat diet for 3 weeks prior to randomization to study groups. On day -1, mice had body composition measured. On day 0, baseline body weight (47.9 g ± 0.2) and food intake were recorded. Mice were randomized to four treatment groups (n = 8/group) to receive either vehicle (PBS), or MEDI4166 (3, 10 or 30 mg/kg), dosed subcutaneously once per week. Body weight and food intake were recorded three times per week. On day 26, mice were subjected to a final body composition assessment. Mice were fasted overnight before recording glucose on day 28 then euthanized by CO_2_ inhalation and terminal blood collected via cardiac puncture to analyse clinical chemistry parameters.

### Repeat-dose study in diabetic *db/db* mice

Male *db/db* (BKS.Cg-m+/+Leprdb/J) mice (Charles River; 5–6 weeks old) were group housed and acclimatized for 34 days with *ad libitum* access to normal chow prior to the study. On day -6, mice were randomized into four groups (n = 12/group): vehicle (PBS), or MEDI4166 (3, 10 or 30 mg/kg). Vehicle or MEDI4166 was dosed subcutaneously once weekly. Blood samples for measuring 4 h fasting blood glucose were collected at treatment day -1, 8, 16, 22, and 27. An IPGTT was performed on study day 22 at around 24 h after dosing.

### Single-dose study in lean cynomolgus monkeys

Male cynomolgus monkeys (2 to 5 years old and weighing between 2.1 to 3.5 kg) were group housed (3 per cage) and acclimatized for at least 4 weeks prior to the study. Animals were allowed access to water *ad libitum* and fed on a standard dry diet (Formula 2050 containing 20% crude protein, 5.4% fat, 40.1% carbohydrate, 8.1% crude fiber, 18.4% neutral detergent fiber and 6.1% ash - Teklad Diets, Madison WI) supplemented with biscuits and fresh fruits. Bodyweight was recorded twice a week during the pre-treatment period, daily during the first week of the treatment and twice a week thereafter. Food consumption was recorded daily. On day 0, animals (n = 3/groups) received a single intravenous (i.v.) dose of MEDI4166 at 1 or 5 mg/kg or a single subcutaneous (s.c.) dose of MEDI4166 at 10 or 100 mg/kg. Blood samples were taken at the following times in relation to dosing: Predose (day 0) for all 4 groups then 5 min, 6 h, 1, 3, 5, 6, 7, 8, 9, 10, 12 and 30 days for the 1 mg/kg (i.v.) group; 5 min, 6 h, 1, 3, 5, 7, 9, 12, 18, 24, 27 and 30 days for the 5 mg/kg (i.v.) group; 2 h, 6 h, 12 h, 1, 3, 5, 6, 8, 12, 18, 24 and 30 days for both the 10 mg/kg and 100 mg/kg (s.c.) groups. Blood samples were processed to either plasma (LDL-C analysis) or serum (human Fc, free PCSK9 and GLP-1R active compound analysis).

### Pharmacokinetic studies

Human antibody Fc concentrations were quantified by a generic sandwich immunoassay method on the Gyrolab platform. Compounds and standards were captured by a biotinylated monoclonal anti-human IgG1 antibody at 100 µg/mL (in-house) and detected by an Alexa-labelled monoclonal anti-human IgG1 antibody at 10 nM (The Binding Site) on a Gyrolab Bioaffy 200 CD platform (Gyros AB). Analysis was performed using the Gyrolab Evaluator software.

Concentrations of GLP-1R active compound were measured using an *ex-vivo* cAMP cell-based assay. Dosing solutions at known concentration were spiked into plasma or serum and used as standard. Samples and standards were serially diluted in assay buffer and tested for cAMP accumulation in CHO cells expressing human GLP-1R as described above. Concentration values were back calculated based on the standard signals and PK parameters were estimated by non-compartmental analysis using Phoenix WinNonlin version 6.3 (Certara). GLP-1R active compound concentrations in cynomolgus monkey serum samples were normalised for Fc concentrations at the first time-point.

### Plasma LDL-cholesterol and free PCSK9 measurement in cynomolgus monkey

Free PCSK9 levels in cynomolgus monkey serum were quantified by a sandwich immunoassay method on the Gyrolab platform. Free PCSK9 in standards, controls and samples was captured by a biotinylated monoclonal anti-human PCSK9 antibody at 100 µg/mL (in-house) and detected by an Alexa-labelled PCSK9 Affinity Purified PAb antibody at 30 nM (R&D Systems) on a Gyrolab Bioaffy 1000 CD platform (Gyros AB). Analysis was performed using the Gyrolab Evaluator software.

Plasma LDL-C levels were conducted by the Department of Biomarkers, Bioanalysis and Clinical Sciences, Envigo, on a Roche P Modular Analyser (Roche Diagnostics).

### Statistical analysis

Graphical presentations, calculations and statistical analyses were carried out with GraphPad Prism software (San Diego, CA, USA). Results are expressed as mean ± standard error of the mean (SEM) unless otherwise stated. *In vivo* data were analysed with a two-way ANOVA followed by Tukey’s post-hoc test (fasted glucose levels, ipGTT glucose excursion, body weight and food intake) or a one-way ANOVA followed by Dunnett’s or Tukey’s post-hoc test (glucose AUC, terminal fat and clinical chemistry data). In all cases, p < 0.05 was considered significant.

## Electronic supplementary material

Supplementary Information

## Data Availability

All data supporting the findings of this study are available within the article and its Supplementary Information file, or are available from the authors upon reasonable request.
