# Supplementary material for: Engineering of a GLP-1 analogue peptide/anti-PCSK9 antibody fusion for type 2 diabetes treatment
Source: Sci Rep. 2018 Dec 3;8:17545. doi: 10.1038/s41598-018-35869-4 (PMC6277417; doi:10.1038/s41598-018-35869-4)
Supplement: Supplementary file 1 — Supplementary Information [file 41598_2018_35869_MOESM1_ESM.pdf]

Engineering of a GLP-1 analogue peptide/anti-PCSK9 antibody fusion for type 2 diabetes treatment

Authors

Matthieu Chodorge, Anthony J. Celeste, Joseph Grimsby, Anish Konkar, Pia Davidsson, David Fairman, Lesley Jenkinson, Jacqueline Naylor, Nicholas White, Jonathan C. Seaman, Karen Dickson, Benjamin Kemp, Jennifer Spooner, Emmanuel Rossy, David C. Hornigold, James L. Trevaskis, Nicholas J. Bond, Timothy B. London, Andrew Buchanan, Tristan Vaughan, Cristina M. Rondinone & Jane K. Osbourn

Supplementary Methods

LC-MS/MS analysis of *in vivo* peptide degradation

Three male Wistar rats (Charles River) received a single i.v. administration at 3 mg/kg of the DPP4 resistant GLP-1 peptide in light chain fusion with a control antibody. Blood samples were taken 7 days post-injection, pooled and processed to plasma. For the reference sample, peptide antibody fusion was diluted at 60 nM into rat plasma (in-house). Fusion protein was extracted using an anti-human Fc capture antibody immobilised on magnetic beads. Approximately 3.0 and 4.5 µg of protein was recovered from spiked and *in vivo* samples respectively and reconstituted in 50 µL of 50 mM ammonium bicarbonate. Extracted protein was reduced by incubation with 10 mM DTT for 60 min at 60°C, alkylated by incubation with 10 mM iodoacetamide at room temperature and then digested by overnight incubation at 37°C with trypsin at approximately 10:1 analyte:enzyme ratio. The resulting peptides were then analysed by LC-MS/MS. The quantity of each tryptic peptides spanning the GLP-1 analogue and linker region and degradant products thereof was determined using a selective

reactive monitoring (SRM) approach. Relative abundances of the different peptides between the spiked and *in vivo* samples were calculated after normalisation to a light chain peptide (T4) that served as control for recovery of fusion protein from plasma. SRM analysis was conducted using an Acquity UPLC (Waters) with a HSS T3 C18 2.1 x 100 mm UPLC column (Waters) and a 5500 triple quadrupole mass spectrometer (Applied Biosystems). SRM transitions were used for quantitation and validated using tandem mass spectrometry.

Rodent pharmacokinetic studies

Pharmacokinetics and in vivo stability of early peptide antibody fusions in mice

Healthy female C57Bl/6 mice (Charles River; 7-8 weeks of age) with a body weight of 20-22g were singly housed upon arrival and allowed to acclimate for 2 weeks. Mice (n=3/time-point/compound) were administered intravenously with peptide antibody compounds Ab#2_GLP1 at 5 mg/kg, Ab#2_EX4 at 1 mg/kg, Ab#2_GLP1 W25K at 5 mg/kg, Ab#2_GLP1 W25N/V27S at 40 mg/kg or Ab#2_DSB#1 at 10.8 mg/kg with a dosing volumes of 10-16 mL/kg. Mice were sacrificed and blood samples were collected in EDTA tubes containing 1:100 v/v of DPP4 inhibitor at the following times post-dosing: 5 min, 6.5 h, 1, 3 and 7 days for Ab#2_GLP1, Ab#2_GLP1 W25K, Ab#2_GLP1 W25N/V27S and 5 min, 6 h, 2, 3 and 7 days for Ab#2_EX4 and Ab#2_DSB#1. Plasma samples were analysed for human Fc concentration using the Gyros platform and GLP-1R active compound concentration using the *ex vivo* cAMP accumulation cell-based assay both as described in the Material & Methods section. PK parameters were determined by linear one- or two-compartment PK analysis using WinNonlin Professional version 5.2 (Pharsight Corp., Mountain View, CA).

Pharmacokinetics and in vivo stability of peptide antibody fusions in rat

Male CD rats weighing 200–250 g at the start of the study (Charles River, UK) were housed under standard conditions with a 12 h light/dark cycle and *ad libitum* access to food and water. Groups of

three animals per compound were injected with Ab#2_DSB#7 at 10 mg/kg i.v. or Ab#2.1_DSB#7_V19A at 60 mg/kg s.c. Blood samples were taken in tubes containing 1:100 v/v of DPP4 inhibitor (Millipore) at the following time-points post-injection: 30 min, 6 h, 1, 2, 4 and 10 days for Ab#2_DSB#7 and 6 h, 1, 2, 4, 7 and 10 days for Ab#2.1_DSB#7_V19A. Samples were processed to serum and analysed for human Fc and GLP-1R active compound concentrations as previously described.

Pharmacokinetics in rat of Ab#2.1_DSB#7 peptide antibody fusion point mutants and anti-PCSK9 mAb Ab#2.1

CD rats were administered i.v. (3 animals per compound) with peptide antibody fusion Ab#2.1_DSB#7_G2V at 60 mg/kg, Ab#2.1_DSB#7_E15A at 53 mg/kg, Ab#2.1_DSB#7_V19A at 58.5 mg/kg or Ab#2.1_DSB#7_L26I at 60 mg/kg at day 0. Samples were taken at the following time post-injection: 30 min, 6 h, 1, 2, 4, 7, 10 and 13 days and processed to serum for human Fc analysis. In a separate experiment, animals (3 per compound) received a single administration at 60 mg/kg i.v. of peptide antibody fusion Ab#2.1_DSB#7_V19A or anti-PCSK9 Ab#2.1 and blood samples were taken 30 min, 1, 3, 7, 14 and 21 days post-dose for human Fc analysis.

Aggregation of peptide antibody fusions

Compounds in 25 mM histidine buffer, 205 mM sucrose (pH 6.0) were concentrated using Amicon Ultra-15 centrifugal filters to achieve a target concentration of 50 mg/mL. Samples were then spiked with 0.02% Polysorbate 80, filtered on 0.2 μm membrane, and 1 mL aliquots placed at 5°C for 4 weeks in type I borosilicate glass vials. Aggregation rates were then determined by size exclusion chromatography on a TSK gel G3000 SWXL 7.8 X 300 mm column after diluting the samples to 10 mg/mL in PBS and filtering on a 0.45 μm membrane.

Affinity parameters for PCSK9 binding

Kinetic and affinity parameters for the binding of antibody and peptide antibody fusions to recombinant PCSK9 were determined by surface plasmon resonance (SPR) using a Biacore 2000 biosensor (GE Healthcare). An anti-human IgG surface was first created using the Human Antibody Capture Kit and CM5 sensor chip (GE Healthcare). Tested compounds at 1 nM were then flowed at 10 µL/min for 3 min. Human, cynomolgus (both in house) or rat PCSK9 (SinoBiological) diluted in running buffer (10 mM sodium phosphate (pH 7.4), 150 mM sodium chloride, 1 mg/mL BSA and 0.05% Tween 20) were injected for 10 min followed by a 10 min dissociation time period. Global dissociation rates were first calculated followed by global on-rate calculations both using a 1:1 binding kinetics model.

Biochemical PCSK9/LDL receptor inhibition assay

Biotinylated human PCSK9 (in-house) was incubated for 2 h with serial dilution of tested compounds in MaxiSorb plate (NUNC) coated with human LDL receptor (R&D Systems). Bound PCSK9 was detected using cryptate labelled streptavidin (Perkin Elmer) diluted at 100 ng/mL in Delfia Buffer (Perkin Elmer). Fluorescence signal was read on a Perkin Elmer Envision machine using a 340 nm excitation and 620 nm emission. Percentage of specific binding was calculated by subtracting the background signal obtained with no LDL receptor coated onto the plate normalized with the maximum specific binding signal obtained with no competitor compound minus background level.

Activity at human receptors closely related to GLP-1R

Activity of MEDI4166 at glucagon, glucagon-like peptide-2 (GLP-2), secretin and gastric inhibitory polypeptide (GIP) receptors was assessed in the cAMP accumulation assay previously described using stable cell lines expressing the different receptors (CHO derived cells for glucagon, GLP-2 and secretin receptors and HEK derived cells for GIP receptor). Specific agonist peptides for each of the four receptors, all purchased from Bachem, were used as positive controls: human glucagon peptide, human GIP peptide, human GLP-2 peptide and human secretin peptide.

Glucose-stimulated insulin secretion *in vitro* assay

Rat insulinoma INS1 832/3 cells (Dr. Christopher B. Newgard, Duke University, NC) were seeded at 250,000 cells/well in a poly-D-lysine coated 48-well culture plate and allowed to grow for 2 days. Cells were then rinsed three times with 250 µL of warm Krebs-Ringers media with 2.7 mM glucose. After the final wash, cells were equilibrated for 1 h in the rinse media. After removing the rinse media, treatment media containing 8.3 mM glucose alone or with GLP-1R agonists was applied to the cells for 1 h. Media was then collected, centrifuged at 300xg for 5 min and insulin concentration determined by electrochemiluminescence immunoassay (MesoScale Discovery, Rockville, MD).

Single-dose study in wildtype and GLP-1R knock-out mice

Male wildtype and GLP-1 receptor-deficient (GLP-1RKO) mice on the C57BL6J background were obtained from heterozygous mating pairs (Jackson Laboratory, Bar Harbor, ME), initially derived from breeding pairs obtained from Dr. Daniel Drucker (Scrocchi L. *et al.*, Nat Med 2: 1254–1258, 1996). Mice (8-12 weeks old) were fed on chow diet and maintained in a temperature-controlled environment (21 ± 2°C and 55 ± 20% humidity) on a normal 12 h light/dark cycle. Two days prior to the study, mice were randomized into groups based on 6 h fasted glucose concentrations and body weight. On the day of study, animals were fasted for 2 h prior to subcutaneous administration of control antibody or MEDI4166 at 10 mg/kg. An intraperitoneal glucose challenge was then performed 4 h post-compound administration.

Blood glucose

Whole blood glucose was measured from wildtype, GLP-1RKO and DIO obese mice using a handheld glucometer (Breeze2, Bayer, Pittsburgh, PA), or BIOSEN c-Line glucose meter (EKF-Diagnostics, Germany) in *db/db* mice.

Glucose tolerance test

Following a 6 h fast, wildtype, GLP-1RKO and DIO mice were administered intraperitoneally 1.5 g/kg glucose (5 mL/kg; at t=0 min). In 4 h fasted *db/db* mice, animals received an intraperitoneal glucose load of 1 g/kg glucose (5 mL/kg; at t=0 min). Blood samples were then collected from the tail vein and blood glucose was measured at -240 (pre-compound administration), 0 (pre-glucose administration), 15, 30, 60 and 120 min following glucose administration in wildtype, GLP-1RKO and DIO mice and 0 (pre-glucose administration), 15, 30, 60, 120 and 240 min following glucose administration in *db/db* mice.

Fat and fat free mass

Body composition (fat mass, fat-free mass) was determined using an EchoMRI instrument (Echo Medical Systems, Houston, TX).

Clinical chemistry parameters

Triglycerides, total cholesterol, and ALT from terminal serum samples were determined using an Olympus bioanalyzer (Olympus America Diagnostics, Center Valley, PA). Insulin concentrations were assessed via ELISA (MesoScale Discovery, Rockville, MD).

PKPD modelling and simulation of GLP-1 analogue peptide α-PCSK9 fusion

A two compartments human PKPD model was built using Berkeley-Madonna (version 8.3.14) based on the diagram described below:

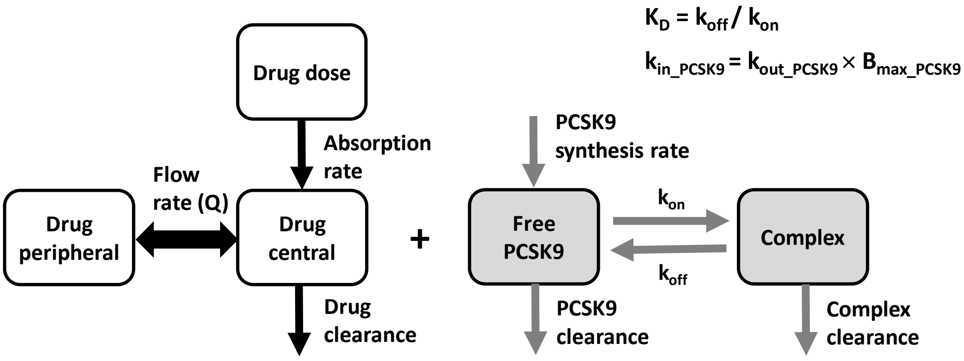

The pharmacokinetic profile of the peptide antibody fusion was assumed to be similar to anti-PCSK9 human IgG molecules. Antibody PK and PCSK9 parameters were obtained from the literature (Lunven C. *et al*., Cardiovasc Ther. (2014): 297-301; Koren M. *et al.,* Postgrad Med. (2015): 125–132; Langslet G. *et al.*, Expert Rev Cardiovasc Ther. (2015): 477-88).

More specifically, bioavailability after subcutaneous administration was set to 80%, rate of absorption to 0.3 day^{-1}, volumes of the central and peripheral compartments to 2 and 3.47 L respectively, flow rate between central and peripheral compartments to 0.33 L/day and central compartment clearance to 0.21 L/day. Plasma PCSK9 concentration was assumed to be 3.4 nM with a clearance rate of 2.76 day^{-1}. Affinity of the compound to human PCSK9 was set to 0.6 nM with an off-rate of 9.7 day^{-1}. As the clearance of human PCSK9 is reported to be faster than that of a typical IgG, target turnover likely drives the clearance of the mAb/PCSK9 complex which has therefore been set up to 0.276 day^{-1}.

The pharmacokinetic profile of the benchmark GLP-1-Fc(γ4) was determined using parameters derived from literature data (Barrington P. *et al.*, Diabetes, Obesity and Metabolism (2011): 434–438). Rate of absorption was set up to 0.48 day^{-1}, central compartment volume to 0.26 L/kg and elimination rate to 0.1752 day^{-1}.

GLP-1 activity profile was generated by normalising the exposure profile by the compound potency at human GLP-1R in the cAMP accumulation assay using CHO transfected cells.

Supplementary Figures

a

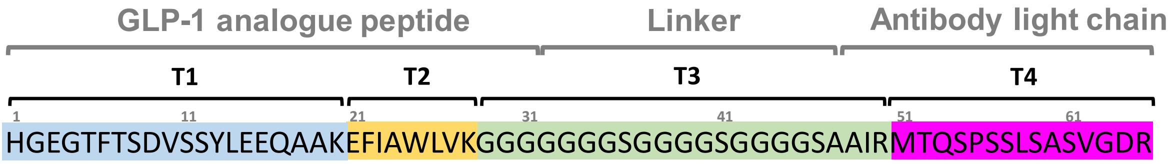

b

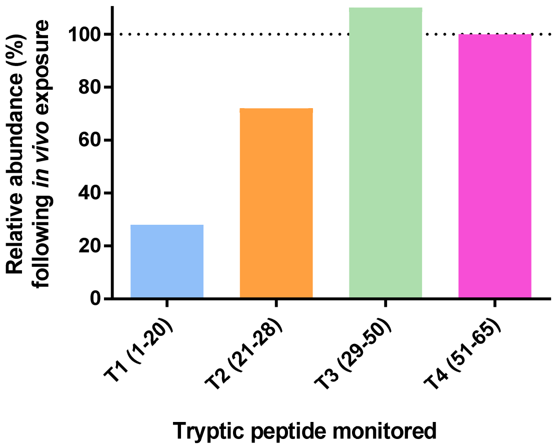

c

SRM peptide			Normalised Counts	
Sequence	Name	Residue	Reference	Sample
EGTFTSDVSSYLEEQAAK	T1*[a]	# 3-20	0	473
YLEEQAAK	T1*[b]	# 11-20	118	417
LVK	T2*	# 25-28	6209	71418

d

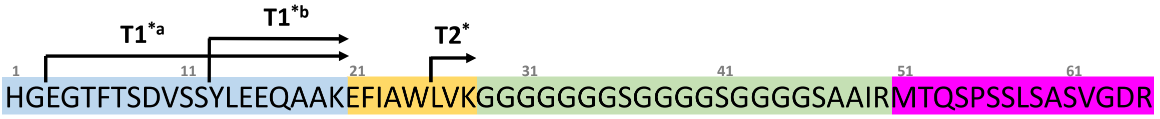

Supplementary Figure 1. ***In vivo*** **degradation of GLP-1 analogue peptide antibody fusion.** (**a**) Amino acid sequence of the GLP-1 analogue antibody light chain fusion showing the first four tryptic peptides spanning the GLP-1 analogue (T1, T2), the linker (T3) and the N-terminus of the light chain (T4). (**b**) Relative abundance of tryptic peptides, determined by LC-MS/MS, after 7 days in rat (sample) compared to compound spiked in plasma (reference). Differences in recovery were normalised using the T4 peptide. (**c**) Selective reaction monitoring (SRM) transitions were used to

identify site specific degradants generated by cleavage within the T1 and T2 peptides. Increase in the relative abundance of T1*[a], T1*[b] and T2* peptides post *in vivo* exposure revealed (**d**) cleavage after G2, S12 and W25.

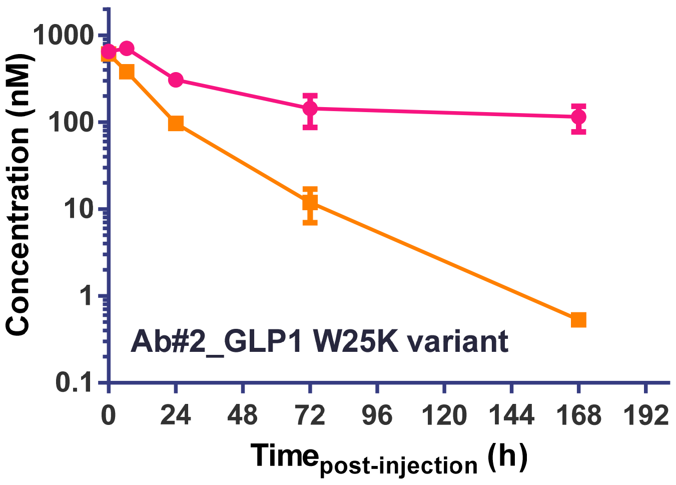

Supplementary Figure 2. Mutating W25 of the peptide component did not prolong *in vivo* GLP-1 activity of the peptide antibody fusion. Human Fc concentration (pink) and GLP-1R active compound concentration (orange) following single intravenous administration in C57Bl/6 mice (n=3 per time-point) of Ab#2_GLP1 W25K variant at 5 mg/kg. Values are presented as mean (± SEM). All other expressed W25 GLP-1 peptide antibody variants (W25R, W25L, W25T and W25A) were truncated on production as assessed by SDS-PAGE and were not further characterized.

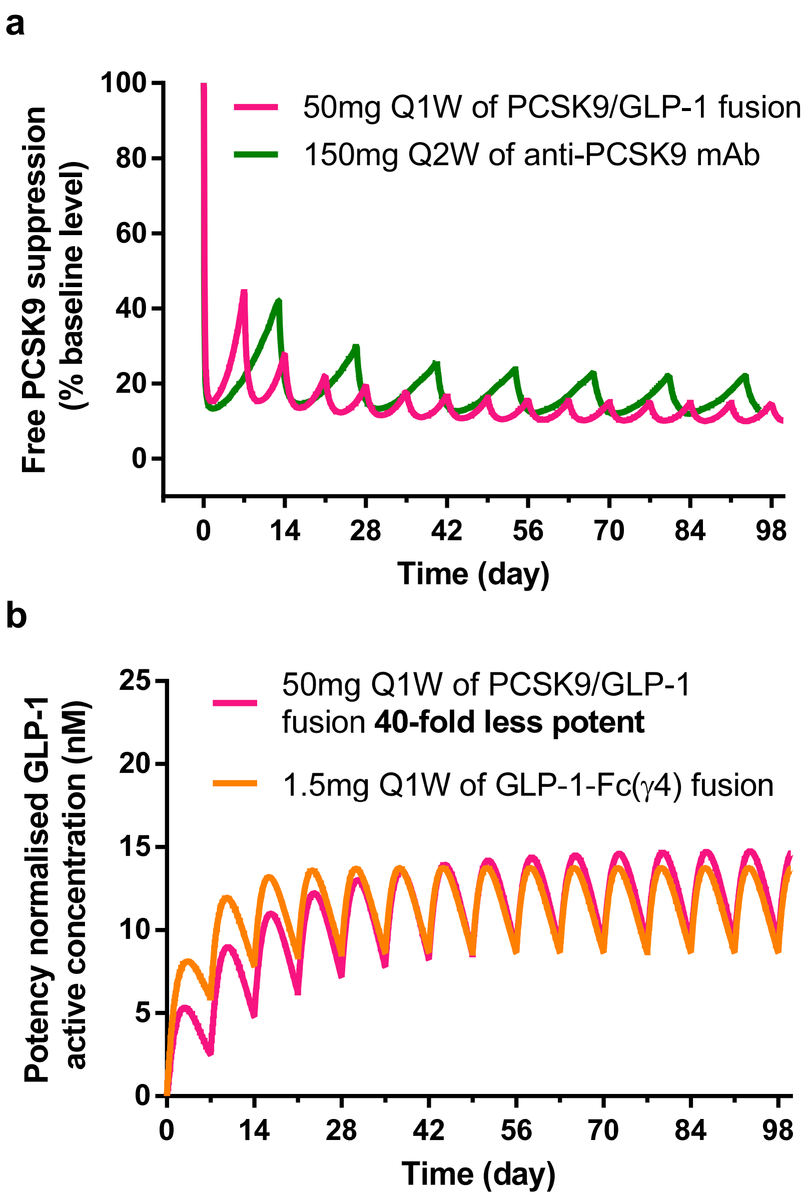

Supplementary Figure 3. PK-PD simulations in human of GLP-1 analogue peptide anti-PCSK9 antibody fusion. (**a**) Free PCSK9 suppression following subcutaneous injection every week of 50 mg peptide antibody fusion or every two weeks of 150 mg anti-PCSK9 mAb. (**b**) Concentration in potency normalised activity at GLP-1R following weekly injection of 1.5 mg GLP-1-Fc(γ4) or 50 mg peptide antibody fusion with a 40-fold reduction in GLP-1R potency compared to the reference molecule GLP-1-Fc(γ4).

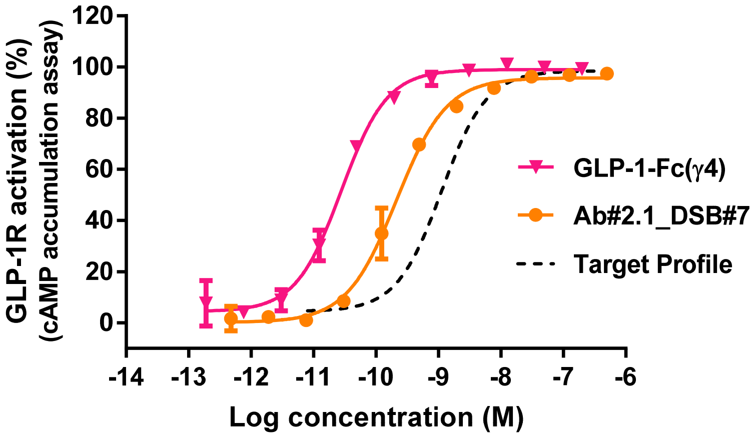

Supplementary Figure 4. Potency at human GLP-1R of the disulphide bridge stabilised GLP-1 analogue peptide DSB#7 in fusion with the antibody Ab#2.1. Activation of human GLP-1R as % of maximum GLP-1-Fc(γ4) response in CHO transfected cells using cAMP accumulation assay following treatment with Ab#2.1_DSB#7 (orange) and GLP-1-Fc(γ4) (pink), (n=2). Values are presented as mean (± SD) of a typical experiment performed independently in duplicate. Predicted activation profile for a molecule exhibiting a 40-fold reduction in potency compared to GLP-1-Fc(γ4) is shown in black dashed line.

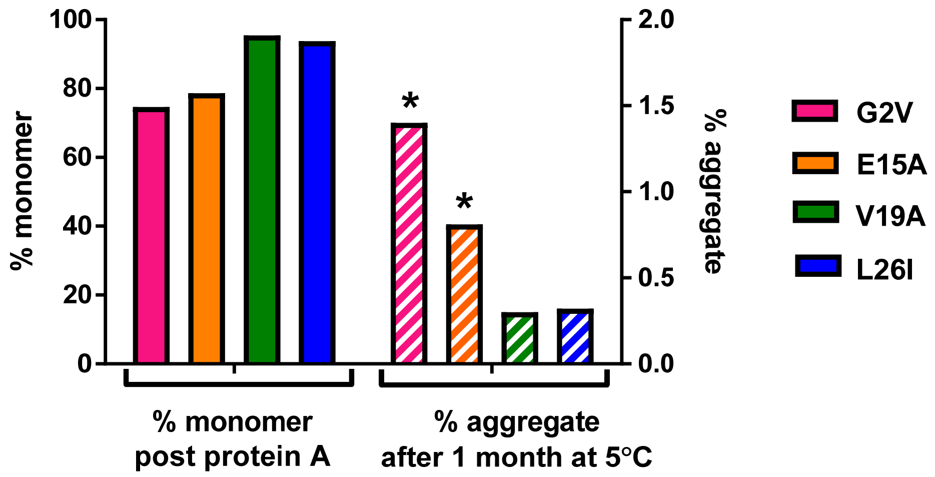

Supplementary Figure 5. Aggregation propensity of GLP-1 analogue peptide anti-PCSK9 antibody fusions. The four selected fusions with the optimised antibody Ab#2.1 and carrying a single amino acid mutation in the DSB#7 peptide moiety to reduce potency at human GLP-1R (G2V (pink), E15A (orange), V19A (green) or L26I (blue)) were analysed for their monomer content following protein A purification (left) and aggregation following a +5°C incubation at high concentration for 4 weeks (right). Compounds were concentrated to around 50 mg/mL except G2V and E15A variants which were concentrated to 39 and 34 mg/mL respectively (*). Percentage in monomer and aggregates were determined by analytical size exclusion chromatography (n=1).

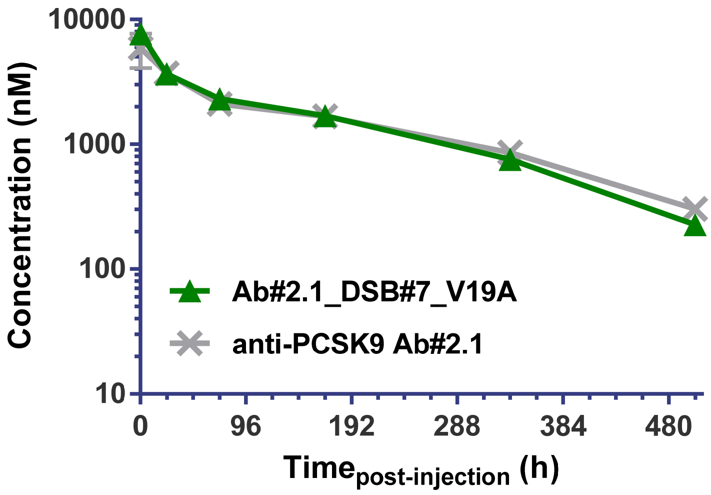

Supplementary Figure 6. Comparison of the PK profile of GLP-1 analogue peptide antibody fusion with anti-PCSK9 mAb. Human Fc concentration following single intravenous administration in CD rats (n=3 per compound) at 60 mg/kg of DSB#7 peptide antibody variant V19A (green) and the corresponding anti-PCSK9 antibody Ab#2.1 (grey). Values are presented as mean (± SEM).

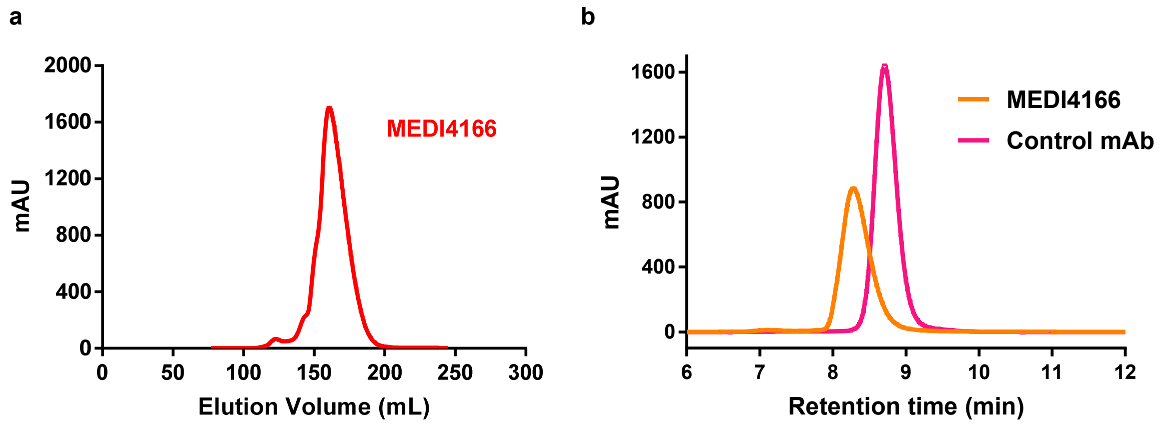

Supplementary Figure 7. MEDI4166 aggregation profile during production. (**a**) Elution profile of preparative size exclusion for MEDI4166 following protein-A purification. (**b**) MEDI4166 analytical SEC-HPLC chromatogram post-purification. An irrelevant human IgG1 mAb was run in parallel as control.

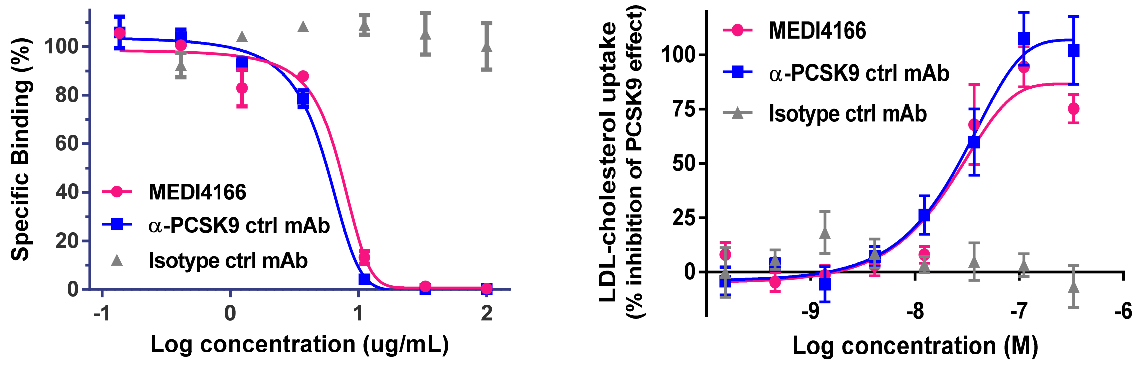

Supplementary Figure 8. ***In vitro*** **characterisation of MEDI4166 for human PCSK9 inhibition.** GLP-1 analogue peptide anti-PCSK9 antibody fusion MEDI4166 (pink), positive control anti-PCSK9 mAb (blue) and an isotype control mAb (grey) were characterised (**a**) for blocking the binding of recombinant human PCSK9 to LDL receptor in a biochemical competition assay (n=3) and (**b**) for restoring the uptake of fluorescently labelled LDL-C by HepG2 cells treated with recombinant human PCSK9 (n=5). Values are presented as mean (± SEM).

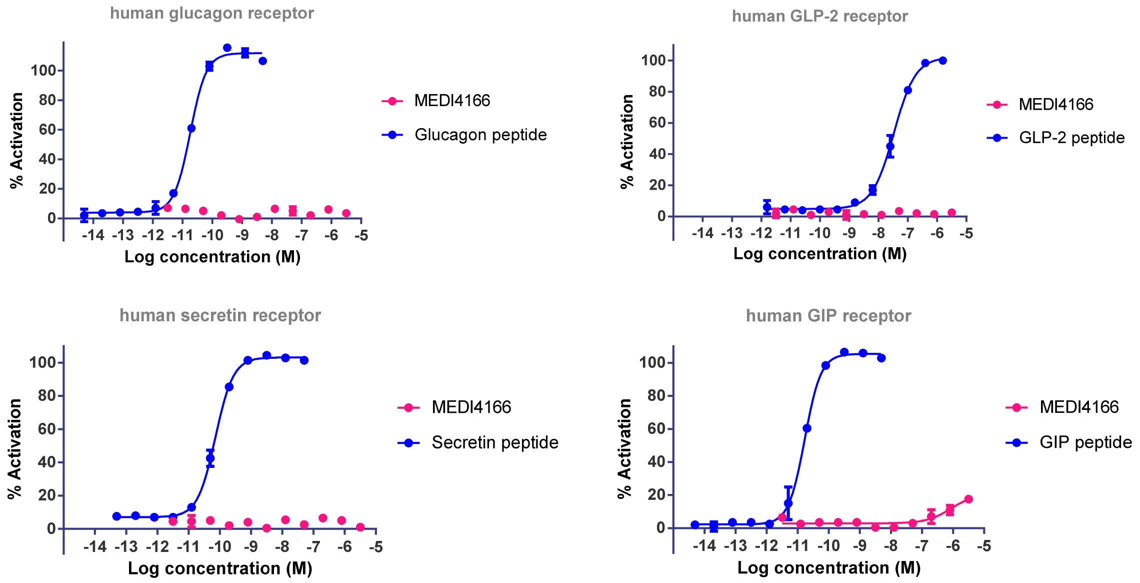

Supplementary Figure 9. Cross-reactivity of MEDI4166 at GLP-1R related human receptors.

Specificity of MEDI4166 for GLP-1R was assessed in cAMP assays using stable CHO cells expressing **(a)** human glucagon receptor (GCGR), **(b)** human glucagon like peptide 2 receptor (GLP-2R) or **(c)** human secretin as well as HEK293 cells expressing **(d)** human GIP receptor (GIPR). Specific agonist peptides for each of the four receptors were used as positive controls: human glucagon peptide, human GIP peptide, human GLP-2 peptide and human secretin peptide. Values are presented as mean (± SD) of n=2 individual experiments.

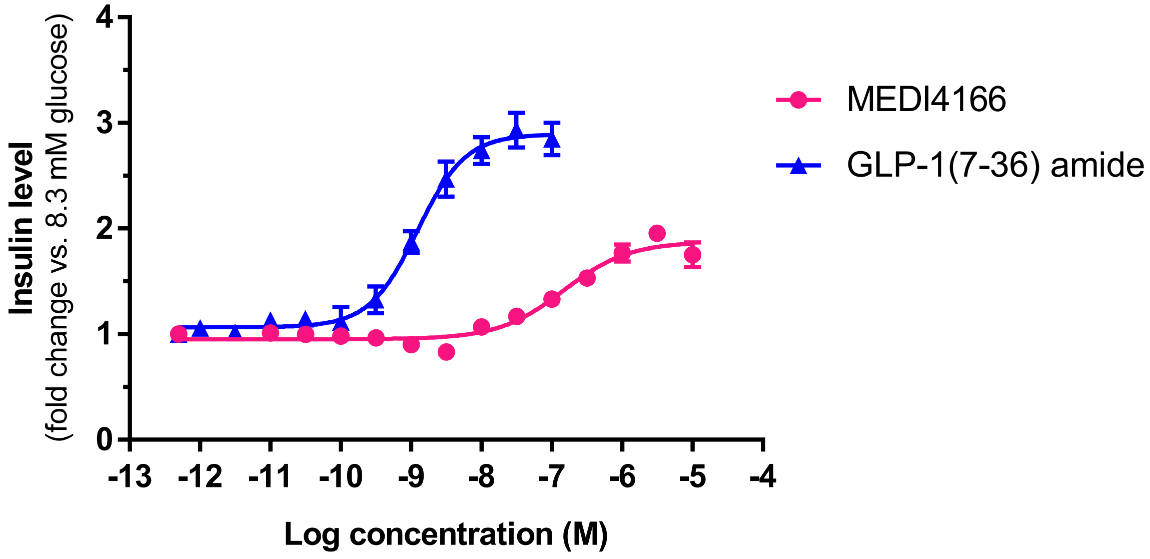

Supplementary Figure 10. Effect of MEDI4166 on glucose-stimulated insulin secretion in INS1 cells. GLP-1(7-36) amide was used as positive control. Insulin levels were normalized to the high glucose (8.3 mM) alone group, and expressed as fold-change. Values are presented as mean (± SEM) from n=6 independent experiments.

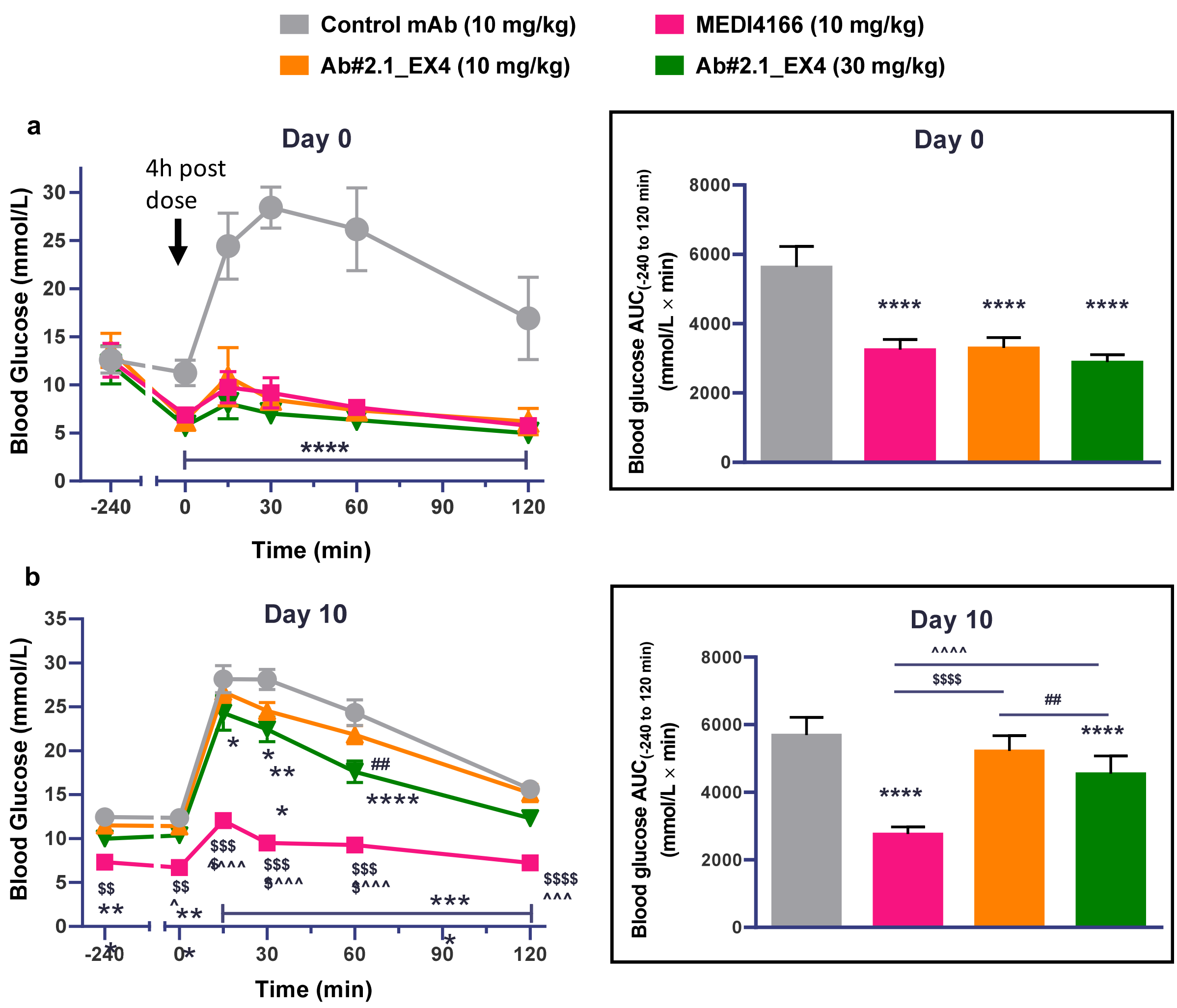

Supplementary Figure 11. Effect over time of a single administration of MEDI4166 on glucose excursion in DIO mice . Glucose excursion measured during an intraperitoneal glucose tolerance test (ipGTT) on (**a**) day 0 and (**b**) day 10 after single subcutaneous administration of anti-PCSK9 control antibody Ab#2.1 at 10 mg/kg (grey), MEDI4166 at 10 mg/kg (pink) or Ab#2.1_EX4 at 10 (orange) or 30 mg/kg (green) to 6 h fasted male DIO mice (n=7-10/group). For both GTT's, glucose load was administered at time 0. For day 0 GTT, glucose was administered 4 h after compound injection. Overall glucose area under the curve (AUC) values are shown in the insets. Values are presented as mean (±SEM). In all cases, *p<0.05; ***p <0.001; ****p<0.0001 compared to isotype control by one-way (AUC-glucose) or two-way (glucose excursion) analysis of variance, followed by Dunnett's or Tukey's *post hoc* test); $^{\$\$}$p<0.01; $^{\$\$\$\$}$p<0.0001 MEDI4166 compared to 10 mg/kg

Ab#2.1_EX4; ^p<0.05; ^^^p<0.001; ^^^^p<0.0001 MEDI4166 compared to 30 mg/kg Ab#2.1_EX4;

##p<0.01 30 mg/kg Ab#2.1_EX4 compared to 10 mg/kg Ab#2.1_EX4.

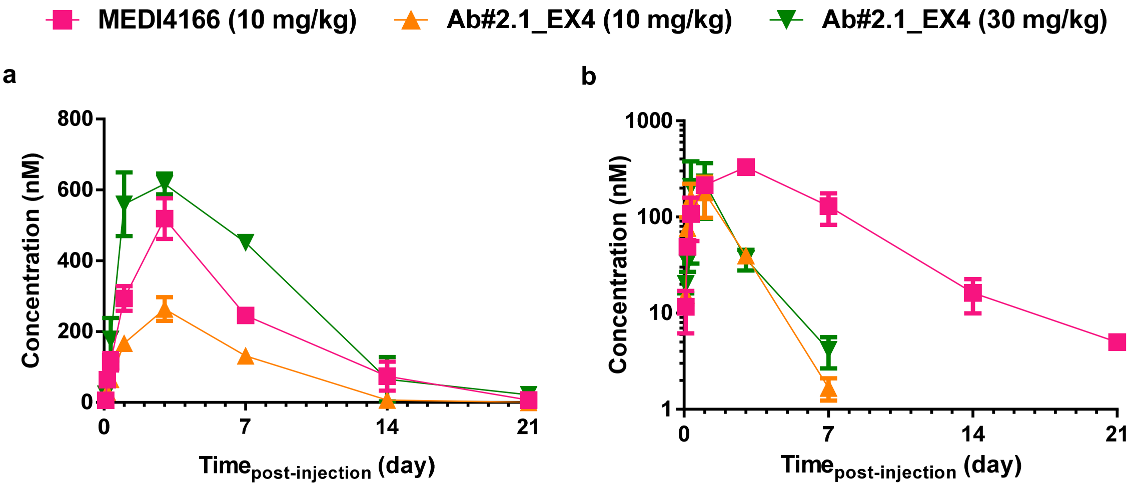

Supplementary Figure 12. Exposure profiles following single administration into DIO mice. (**a**) Human Fc concentration and (**b**) GLP-1R active compound concentration after single subcutaneous administration of MEDI4166 at 10 mg/kg (pink) or Ab#2.1_EX4 at 10 (orange) or 30 mg/kg (green) to male C57Bl/6 DIO mice (n=3/group). Data are shown as mean (± SEM).

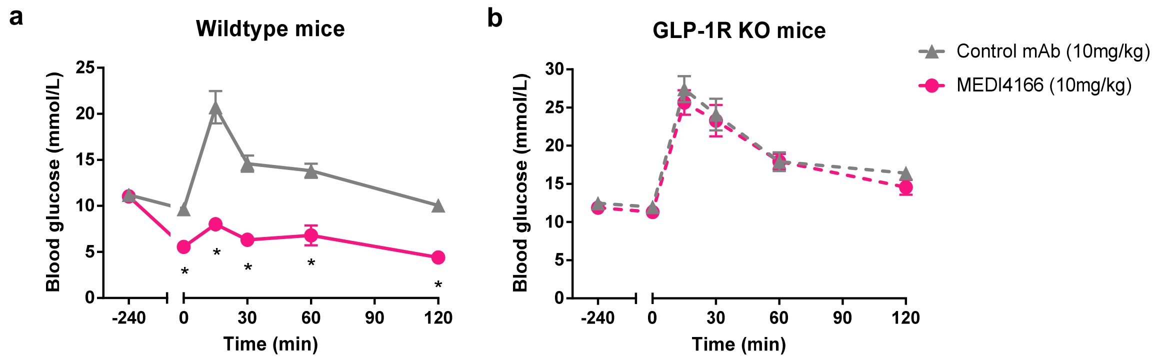

Supplementary Figure 13. Effect of MEDI4166 on blood glucose appearance in wildtype and GLP-1R knockout mice after intraperitoneal glucose challenge. Control antibody (10 mg/kg) or MEDI4166 (10 mg/kg) were administered subcutaneously to **(a)** lean C57Bl/6 wildtype or **(b)** GLP-1R knockout (GLP-1R KO) mice 4 h prior to an intraperitoneal injection of glucose (1.5 g/kg; n=7-8/group). Values are presented as mean (± SEM). *p<0.05 vs. vehicle.

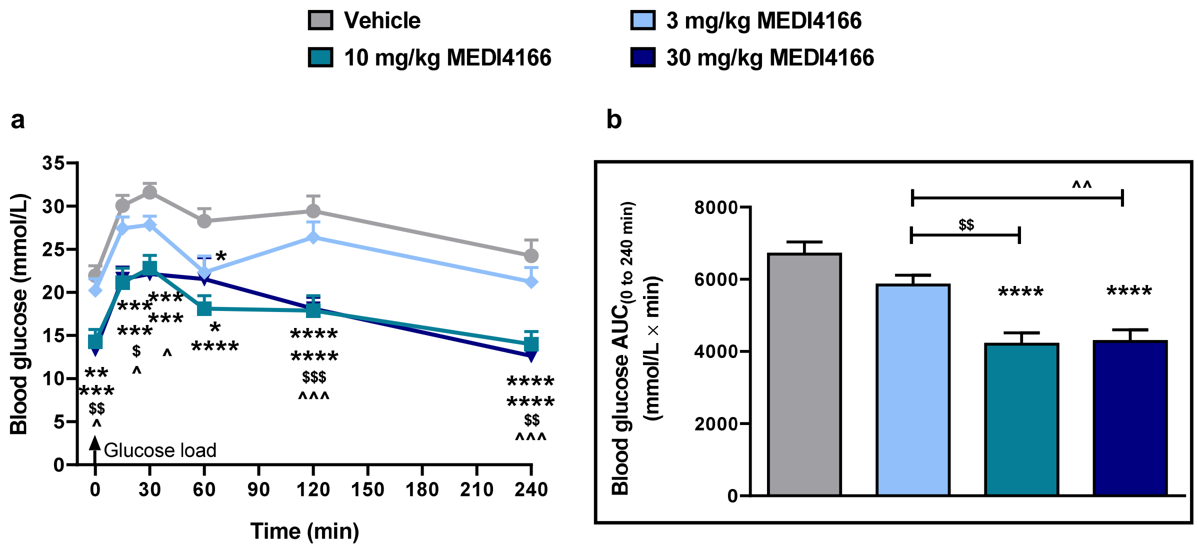

Supplementary Figure 14. Effect of MEDI4166 on glucose excursion following repeated administration into diabetic *db/db* mice. (**a**) Glucose excursions observed in an ipGTT performed on day 22 after weekly subcutaneous administration of vehicle (PBS) or MEDI4166 at 3, 10 or 30 mg/kg in 4 h fasting male diabetic *db/db* mice done by administration at time t=0 min of 1 g/kg body weight of glucose (n=12/group). (**b**) Corresponding overall glucose area under the curve (AUC) values. Data are presented as mean (± SEM);**p < 0.05; ***p< 0.001; ****p<0.0001 compared to vehicle by one-way (AUC-glucose) or two-way (glucose excursion) analysis of variance, followed by Tukey's *post hoc* test; $^{\$}$p<0.05; $^{\$\$}$p<0.01; $^{\$\$\$}$p<0.001 3 mg/kg MEDI4166 compared to 10 mg/kg MEDI4166; ^p<0.05; ^^p<0.01; ^^^p<0.001; ^^^^p<0.0001 3 mg/kg MEDI4166 compared to 30 mg/kg MEDI4166.

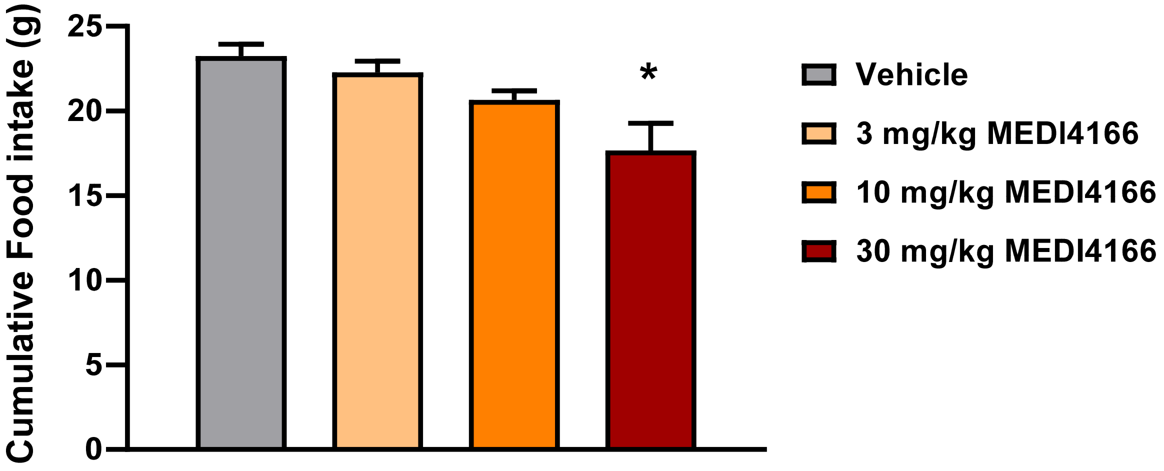

Supplementary Figure 15. Effect of MEDI4166 on food intake following repeated administration into DIO mice. Cumulative food intake following once weekly subcutaneous administration of vehicle (PBS) or MEDI4166 at 3 (salmon), 10 (orange) or 30 (dark red) mg/kg for 26 days. Values are presented as mean (± SEM); n = 8/group. *$p < 0.05$ compared to vehicle.

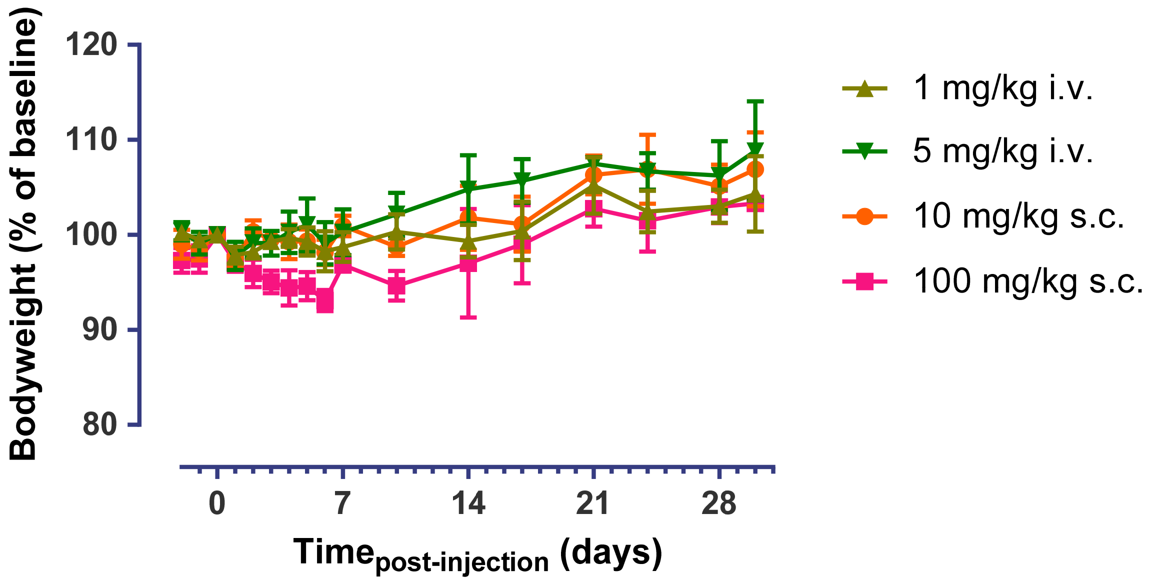

Supplementary Figure 16. Cynomolgus monkey bodyweights following single injection of MEDI4166. Bodyweights in the 4 dosing groups: intravenous administration at 1 (khaki) or 5 (green) mg/kg or subcutaneous administration at 10 (orange) or 100 (pink) mg/kg. Data were normalized to Day 0 values (pre-injection) and expressed as % of baseline. n=3 animals per group. Values are presented as mean (± SEM).

Supplementary Tables

Antibody		Antibody generation strategy
	Ab#1	Humanised antibody from mouse immunisations using recombinant human PCSK9 antigen
	Ab#2	Humanised antibody from mouse immunisations using recombinant human PCSK9 antigen
	Ab#3	Human antibody from humanised mouse immunisations using recombinant human PCSK9 antigen
	Ab#4	Human antibody from humanised mouse immunisations using recombinant human PCSK9 antigen
	Ab#5	Human antibody from humanised mouse immunisations using recombinant human PCSK9 antigen
	Ab#6	Human antibody from phage display libraries selected against recombinant murine PCSK9 antigen
	Ab#7	Human antibody from phage display libraries selected against recombinant human and murine PCSK9 antigens

Supplementary Table 1. Origin of the anti-PCSK9 antibodies. Generation strategy of the seven anti-PCSK9 mAbs used for creating GLP-1 analogue peptide antibody fusions.

Amino acid similarity in V_H antibody sequences (%)

	Antibody						
Antibody	Ab#1	Ab#2	Ab#3	Ab#4	Ab#5	Ab#6	Ab#7
Ab#1		98.3	57.5	54.2	52.5	75.4	63.8
Ab#2			57.5	54.2	52.5	75.4	63.8
Ab#3				77.1	72.0	53.4	55.2
Ab#4					78.0	51.2	54.6
Ab#5						49.2	50.4
Ab#6							65.5
Ab#7							

Amino acid similarity in V_L antibody sequences (%)

	Antibody						
Antibody	Ab#1	Ab#2	Ab#3	Ab#4	Ab#5	Ab#6	Ab#7
Ab#1		71.0	70.1	90.3	52.3	72.3	72.6
Ab#2			93.5	69.2	51.9	57.0	84.0
Ab#3				70.1	52.8	55.1	79.2
Ab#4					51.4	69.6	69.8
Ab#5						46.8	51.4
Ab#6							61.3
Ab#7							

Supplementary Table 2. Anti-PCSK9 antibody sequence homology. Amino acid percentage identity for the variable heavy (left) and light (right) chains across the seven selected anti-PCSK9 mAbs.

Potency at human GLP-1R in cAMP assay (M)

Fusion to:	VH		VL	
**Linker:**	**Short**	**Long**	**Short**	**Long**
**Ab#1**	nd	2.0E-10	2.4E-09	1.7E-10
**Ab#2**	1.8E-09	2.6E-10	8.3E-09	1.8E-10
**Ab#3**	7.6E-10	1.2E-09	5.0E-10	1.1E-10
**Ab#4**	9.3E-10	7.4E-11	2.3E-09	1.5E-10
**Ab#5**	3.3E-08	6.8E-10	4.1E-10	1.5E-10
**Ab#6**	9.2E-10	1.6E-10	2.4E-10	1.0E-10
**Ab#7**	5.5E-09	7.4E-11	6.5E-09	3.1E-10

Antibody

Relative PCSK9 binding to parental mAb (fold change in IC_{50})

Fusion to:	VH		VL	
**Linker:**	**Short**	**Long**	**Short**	**Long**
**Ab#1**	nd	2.0	2.3	4.0
**Ab#2**	6.1	3.3	3.2	2.2
**Ab#3**	1.4	1.4	4.7	3.1
**Ab#4**	1.8	2.2	1.2	1.6
**Ab#5**	7.1	3.8	12.5	32.3
**Ab#6**	1.1	0.7	101.8	69.0
**Ab#7**	1.8	2.1	121.9	6.3

Antibody

Supplementary Table 3. Activities of GLP-1 receptor agonist peptide anti-PCSK9 antibody fusions.

Potency at human GLP-1R in CHO transfected cells using the cAMP accumulation assay (left) and relative PCSK9 binding compared to the parental anti-PCSK9 mAbs in the biochemical epitope competition assays (right) for the 27 tested peptide antibody fusions. Peptide fusion to the antibody Ab#1 heavy chain with a short linker did not express in sufficient quantity and was not characterised. nd: not determined. Values are presented as mean of n=2.

Compound	Experimental design	GLP-1 Activity Terminal half-life (h)	GLP-1 Activity SD	Fc Exposure Terminal half-life (h)	Fc Exposure SD
Ab#2_GLP1	5 mg/kg i.v. in C57Bl/6 mice - n=3	19.4	1.0	81.7	7.3
Ab#2_GLP1 W25K	5 mg/kg i.v. in C57Bl/6 mice - n=3	18.7	1.8	>100 *	/
Ab#2_GLP1 W25N/V27S	40 mg/kg i.v. in C57Bl/6 mice - n=3	77.1	10.9	>100 *	/
Ab#2_EX4	1 mg/kg i.v. in C57Bl/6 mice - n=3	11.5	0.5	85.4	2.8
Ab#2_DSB#1	10.8 mg/kg i.v. in C57Bl/6 mice - n=3	82.6	6.0	78.0	10.9
Ab#2_DSB#7	10 mg/kg i.v. in CD rats - n=3	57.9	4.5	>100 *	/
Ab#2.1	60 mg/kg i.v. in CD rats - n=3	nd	nd	136	7.5
Ab#2.1_DSB#7_V19A	60 mg/kg i.v. in CD rats - n=3	nd	nd	124	18.2
Ab#2.1_DSB#7_V19A	60 mg/kg s.c. in CD rats - n=3	54.3	6.1	>100 *	/
MEDI4166	5 mg/kg i.v. in cynomolgus monkeys - n=3	63.3	16.8	51	2.52
MEDI4166	100 mg/kg s.c. in cynomolgus monkeys - n=3	116	11	147	27.3

Supplementary Table 4. Terminal half-life of GLP-1 analogue peptide antibody fusions for GLP-1R active compound and Fc exposure. Terminal half-life was determined by noncompartmental analysis using WinNonlin.* Due to very long half-life (>100 h), most of the PK profile was extrapolated, resulting in failed NCA acceptance criteria. nd: not determined. Doses of the peptide-antibody fusions tested in rodent for stability of GLP-1 activity were adjusted based on GLP-1R potency and material availability.

Peptide name	1	11	21	31	41	51	
Exendin-4	H	G E G T F T S D L S	K Q M E E E A V R L	F I E W L K N G G P	S S G A P P P S		
DSB#1	H	G E G T F T S C L S	K Q M E E E A V R L	F I E W L K N G G P	S S G A P P P S G G	G G G G G G G G G C	G G
DSB#2*	H	G E C T F T S D L S	K Q M E E E A V R L	F I E W L K N G G P	S S G A P P P S G G	G G G G G G G G G G	C G
DSB#3	H	G E G T F T S D L S	K Q M E E E C V R L	F I E W L K N G G P	S S G A P P G C		
DSB#4*	H	G E G T F T S D L S	K Q M E E E A V R C	F I E W L K N G G P	S S G A G G C S		
DSB#5	H	G E G T F T S C L S	K Q M E E E A V R L	F I E W L K N G G P	S S G A P P P S G G	G G G G G G G C G G	
DSB#6	H	G E G T F T S C L S	K Q M E E E A V R L	F I E W L K N G G P	S S G A P P P S G G	G G G G G G G G C G	G G
DSB#7	H	G E G T F T S D L S	K Q M E E E C V R L	F I E W L K N G G P	S S G A P P P G C G		
DSB#8	H	G E G T F T S D L S	K Q M E E E C V R L	F I E W L K N G G P	S S G A P P G G C G		
DSB#9	H	G E G T F T S D L S	K Q M E E E A V R C	F I E W L K N G G P	S S G A P P C G G		
DSB#10	H	G E G T F T S D L S	K Q M E E E A V R C	F I E W L K N G G P	S S G A P P G C G		
DSB#11	H	G E G T F T S D L S	K Q M E E E A V R L	F I E C L K N G G P	S S G A C G G S		
DSB#12	H	G E G T F T S D L S	K Q M E E E A V R L	F I E C L K N G G P	S S G A P C P S		
DSB#13	H	G E G T F T S D L S	K Q M E E E A V R L	F I E C L K N G G P	S S G A P P C S		

Supplementary Table 5. Amino acid sequence of disulphide bridge stabilised exendin-4 peptides. Cysteine residues are shown within highlighted black squares. Glycine residues at the C-terminus of Exendin-4 and before the linker are highlighted in grey. Additional mutations to the cysteine bridge in the exendin-4 sequence are shown in red. *Fusion molecules DSB#2 and DSB#4 did not express and were not further characterised.

Activity at human GLP-1R		
**Point mutation**	**Potency (M)**	**% maximum of GLP-1-Fc(γ4) effect**
G2Y	1.7E-08	10
G2V	1.2E-09	93
G2T	6.3E-09	73
G2Q	1.2E-08	86
G2N	1.9E-08	31
G2I	2.3E-09	87
G2F	5.8E-09	69
E15G	6.8E-09	91
E15A	1.2E-09	92
V19T	4.0E-10	95
V19S	2.7E-09	94
V19G	1.3E-08	93
V19A	1.3E-09	96
I23G	5.6E-07	82
I23A	6.1E-07	67
L26T	1.3E-07	87
L26P	1.4E-06	60
L26N	4.3E-06	44
L26Q	5.9E-06	34
L26M	5.3E-08	90
L26I	7.4E-10	96
L26H	8.0E-08	87
L26G	2.8E-06	81

Supplementary Table 6. Activity at human GLP-1R of DSB#7 peptide point mutants in fusion with the antibody Ab#2.1. Potency and maximum activation compared to GLP-1-Fc(γ4) in the cAMP accumulation assay using CHO transfected cells for the 23 tested single point amino acid variants. Values are presented as mean of n=2.

	Association		Dissociation		
**PCSK9**	**k_a ($M^{-1}.s^{-1}$)**	**Chi^2**	**k_d (s^{-1})**	**Chi^2**	**K_d (nM)**
**Human**	$1.9 \times 10^{+5}$	0.89	1.1×10^{-4}	0.16	0.60
**Cynomolgus**	$1.1 \times 10^{+5}$	1.91	1.9×10^{-4}	0.53	1.70
**Rat**	$3.7 \times 10^{+5}$	0.77	2.1×10^{-4}	0.11	0.57

Supplementary Table 7. Kinetic parameters of MEDI4166 for human, cynomolgus and rat PCSK9. Binding parameters were determined by SPR after capturing MEDI4166 and flowing recombinant PCSK9 at 6 different concentrations. Data were fitted to a 1:1 Langmuir model using separate association and dissociation. Chi^2 values are presenting goodness of fit.

Compound	EC_{50} (M)
**MEDI4166**	$4.3\times10^{-9} \pm 9.9\times10^{-10}$
**GLP-1(7-36) amide**	$2.9\times10^{-11} \pm 4.3\times10^{-12}$
**GLP-1-Fc(γ4)**	$9.5\times10^{-11} \pm 1.8\times10^{-11}$

Supplementary Table 8. MEDI4166 *in vitro* potency at human GLP-1R in the cAMP accumulation assay. Activity of MEDI4166 and positive controls GLP-1(7-36) amide peptide and GLP-1-Fc(γ4) using CHO transfected human GLP-1R. Values are geometric mean (± SEM) from n=7 independent experiments.

Compound	mouse GLP-1R CHO		MIN6 cells	
	EC_{50} (M)	n	EC_{50} (M)	n
**MEDI4166**	$1.1\times10^{-9} \pm 5.4\times10^{-10}$	11	$2.8\times10^{-9} \pm 1.2\times10^{-9}$	9
**Ab#2.1_EXE4**	$2.1\times10^{-11} \pm 1.6\times10^{-11}$	3	$7.5\times10^{-11} \pm 2.1\times10^{-11}$	3

Supplementary Table 9. MEDI4166 *in vitro* potency at mouse GLP-1R in the cAMP accumulation assay. Activity of MEDI4166 and control fusion Ab#2.1_EX4 in CHO cells transfected with mouse GLP-1R and MIN6 insulinoma cells endogenously expressing mouse GLP-1R. Values are geometric mean (± SEM) from n=3-11 independent experiments.

Treatment	Fat mass (g)		Fat free mass (g)	
	Day -1	Day 26	Day -1	Day 26
**Vehicle**	18.4 ± 0.39	18.3 ± 0.77	28.2 ± 0.39	28.0 ± 0.61
**3 mg/kg MEDI4166**	18.0 ± 0.55	18.3 ± 0.45	28.3 ± 0.36	27.9 ± 0.56
**10 mg/kg MEDI4166**	18.3 ± 0.48	17.5 ± 0.47	28.5 ± 0.41	27.2 ± 0.59
**30 mg/kg MEDI4166**	18.1 ± 0.35	15.2 ± 0.91*	28.7 ± 0.34	26.7 ± 0.58

Supplementary Table 10. Effect of repeated MEDI4166 treatment on fat mass in DIO mice. Fat mass and fat free mass as measured by NMR at baseline (day -1) and on day 26 following once weekly subcutaneous administration of vehicle (PBS) or MEDI4166 (3, 10 or 30 mg/kg). Values are presented as mean (± SEM); n = 8/group. *p<0.05 as compared to vehicle.

Treatment	Fasting glucose (mg/dL)	Cholesterol (mg/dL)	Plasma Insulin (ng/mL)	ALT (U/L)
**Vehicle**	159.8 ± 6.2	180.6 ± 11.8	1.16 ± 0.09	132.9 ± 19.1
**3 mg/kg MEDI4166**	99.6 ± 3.1*	178.4 ± 5.1	4.66 ± 1.51	113.9 ± 12.6
**10 mg/kg MEDI4166**	91.1 ± 4.4*	159.0 ± 5.9	1.73 ± 0.40	89.6 ± 7.6
**30 mg/kg MEDI4166**	94.1 ± 3.8*	134.6 ± 7.3**	1.90 ± 1.07	69.7 ± 9.8*

Supplementary Table 11. Effect of repeated MEDI4166 treatment on clinical chemistry parameters in DIO mice. Blood glucose, total cholesterol, plasma insulin and ALT levels on day 28 in overnight fasted mice following once weekly subcutaneous administration of vehicle (PBS) or MEDI4166 (3, 10 or 30 mg/kg). Values are presented as mean (± SEM); n = 8/group. *p<0.05; **p<0.01 as compared to vehicle.
